# Loss of *Prm1* leads to defective chromatin protamination, impaired PRM2 processing, reduced sperm motility and subfertility in male mice

**DOI:** 10.1242/dev.200330

**Published:** 2022-06-17

**Authors:** Gina Esther Merges, Julia Meier, Simon Schneider, Alexander Kruse, Andreas Christian Fröbius, Gregor Kirfel, Klaus Steger, Lena Arévalo, Hubert Schorle

**Affiliations:** 1 Department of Developmental Pathology, Institute of Pathology, University Hospital Bonn, 53127 Bonn, Germany; 2 Department of Urology, Pediatric Urology and Andrology, Section Molecular Andrology, Biomedical Research Center of the Justus-Liebig University, 35392 Giessen, Germany; 3 Department of Molecular Cell Biology, Institute for Cell Biology, University of Bonn, 53121 Bonn, Germany

**Keywords:** Protamine ratio, Protamine 1 (*Prm1*), PRM2 processing, Infertility, Subfertility, Chromatin condensation, Mouse

## Abstract

One of the key events during spermiogenesis is the hypercondensation of chromatin by substitution of the majority of histones by protamines. In humans and mice, protamine 1 (*PRM1*/*Prm1*) and protamine 2 (*PRM2*/*Prm2*) are expressed in a species-specific ratio. Using CRISPR-Cas9-mediated gene editing, we generated *Prm1*-deficient mice and demonstrated that *Prm1*^+/−^ mice were subfertile, whereas *Prm1*^−/−^ mice were infertile. *Prm1*^−/−^ and *Prm2*^−/−^ sperm showed high levels of reactive oxygen species-mediated DNA damage and increased histone retention. In contrast, *Prm1*^+/−^ sperm displayed only moderate DNA damage. The majority of *Prm1*^+/−^ sperm were CMA3 positive, indicating protamine-deficient chromatin, although this was not the result of increased histone retention in *Prm1*^*+/−*^ sperm. However, sperm from *Prm1*^+/−^ and *Prm1*^−/−^ mice contained high levels of incompletely processed PRM2. Furthermore, the PRM1:PRM2 ratio was skewed from 1:2 in wild type to 1:5 in *Prm1*^+/−^ animals. Our results reveal that PRM1 is required for proper PRM2 processing to produce mature PRM2, which, together with PRM1, is able to hypercondense DNA. Thus, the species-specific PRM1:PRM2 ratio has to be precisely controlled in order to retain full fertility.

## Abstract (precis)

**Summary:** Analysis of protamine 1-deficient mice revealed that heterozygous (*Prm1*^+/−^) males are subfertile, whereas homozygous males are infertile, with *Prm1* deficiency leading to defective chromatin compaction and sperm damage.

## INTRODUCTION

During spermatogenesis in the seminiferous epithelium of the testis, diploid spermatogonia differentiate into haploid spermatids. One of the most remarkable changes during spermiogenesis is complete reorganization of chromatin compaction (Rathke et al., 2014), whereby histones are almost completely substituted by protamines. These are highly basic, arginine-rich proteins (Balhorn, 1982) that, upon binding to DNA, hypercondense chromatin, leading to transcriptional silencing and protection of the paternal genome (Steger, 1999). Whereas, in most mammals, DNA compaction in sperm is accomplished by incorporation of protamine 1 (PRM1) alone, primates and most rodents express two protamines, PRM1 and protamine 2 (PRM2) (Chauviere et al., 1992; Retief and Dixon, 1993). In mice and humans, *Prm1* and *Prm2* are encoded in a tightly regulated gene cluster on chromosome 16 (Reeves et al., 1989; Wykes and Krawetz, 2003). Whereas PRM1 is expressed as mature protein, PRM2 is expressed as precursor protein (pre-PRM2), consisting of a C-terminal mature PRM2 (mPRM2) domain and an N-terminal cleaved PRM2 (cPRM2) domain, which is sequentially cleaved off upon binding to DNA (Balhorn, 2007; Retief and Dixon, 1993; Yelick et al., 1987). mPrm2 is proposed to originate from a gene duplication of *Prm1* (Krawetz and Dixon, 1988). In an evolutionary context, *Prm1* and cPrm2 were shown to be conserved, suggesting important roles in fertility (Lüke et al., 2016b; Lüke et al., 2016a). *PRM1*/PRM1 and *PRM2*/PRM2 are detected in a species-specific ratio [1:1 in humans (de Mateo et al., 2009) and 1:2 in mice (Corzett et al., 2002)]. In humans, alterations of this protamine ratio (PRM1:PRM2) have been associated with male sub- and infertility (Aoki et al., 2005; Balhorn et al., 1988; Belokopytova et al., 1993; Bench et al., 1998; de Yebra et al., 1998; García-Peiró et al., 2011; Khara et al., 1997; Ni et al., 2016; Oliva, 2006; Steger et al., 2001; Steger et al., 2003; Steger et al., 2008; Torregrosa et al., 2006).

Mice chimeric for a deletion of one allele of either *Prm1* or *Prm2* (Cho et al., 2001; Cho et al., 2003; Takeda et al., 2016) are infertile and do not allow for the establishment of mouse lines or detailed analysis of Prm deficiency. Furthermore, heterozygous *Prm1*-deficient mice generated with CRISPR-Cas9 are reported to be infertile (Mashiko et al., 2013). Thus, a detailed phenotypical analysis of *Prm1*-deficient mice has not been possible so far.

Schneider et al. reported the establishment of *Prm2*-deficient mouse lines using CRISPR-Cas9-mediated gene editing in zygotes (Schneider et al., 2016). They showed that *Prm2*^+/−^ male mice remained fertile, whereas *Prm2*^−/−^ were infertile. In addition, *Prm2*^+/−^ sperm showed no pathomorphological effects, whereas *Prm2*^−/−^ sperm presented with fragmented DNA, disrupted sperm membranes and complete immotility. These defects were shown to accumulate during epididymal transit. It was also demonstrated that the *Prm2*^−/−^ mice displayed deregulation of proteins, leading to accumulation of reactive oxygen species (ROS), thus explaining the phenotype observed previously (Schneider et al., 2020).

Using CRISPR-Cas9-mediated gene editing in zygotes, we generated mice deficient for *Prm1*. Male mice heterozygous for the mutation (*Prm1*^+/−^) were subfertile, whereas *Prm1*-deficient (*Prm1*^−/−^) males were infertile. Molecular analyses revealed that loss of one allele of *Prm1* led to a moderate fragmentation of DNA, whereas complete DNA fragmentation was observed in *Prm1*^−/−^ mice. Sperm of *Prm1*^+/−^ mice displayed reduced motility as well as enhanced 8-hydroxydeoxyguanosine (8-OHdG) levels, indicative of upregulated ROS levels. Most importantly, analyses of sperm nuclear proteins revealed that the processing of PRM2 to its mPRM2 form already appeared to be disturbed in *Prm1*^+/−^ animals. Furthermore, the species-specific protamine ratio was shifted in *Prm1*^+/−^ mice. These data strongly suggest that the species-specific level of PRM1 is required for proper sperm function.

## RESULTS

### CRISPR-Cas9-mediated gene editing produces *Prm1*-deficient mice

**Fig. 1.**
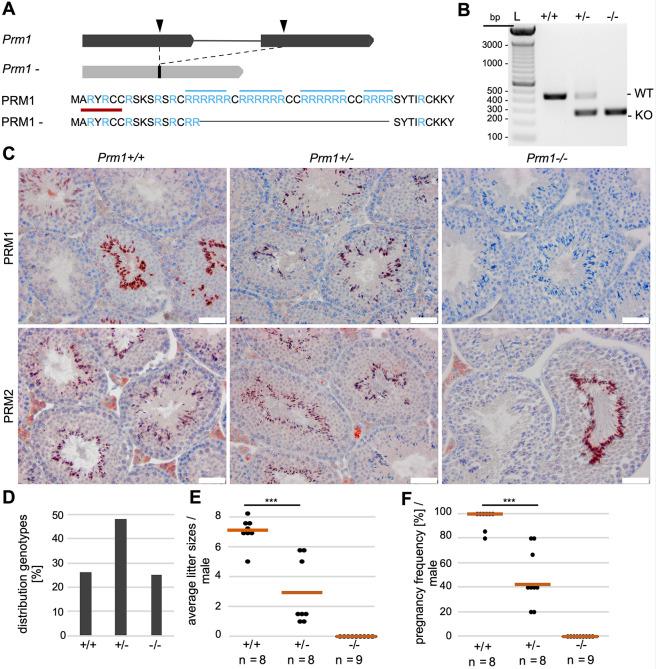
**Establishment of *Prm1*-deficient mice and fertility analysis.** (A) Graphical representation of CRISPR-Cas9-mediated gene editing of the *Prm1* locus. Two sgRNAs were used (black arrowheads), targeting the *Prm1* coding sequence in exon 1 and exon 2, respectively. A 167 bp in-frame deletion was generated, leading to loss of crucial arginine-rich DNA-binding sites (marked in blue). The epitope of the anti-PRM1 antibodies used in (C) is marked in red. (B) Agarose gel of genotyping PCR of *Prm1*^+/+^, *Prm1*^+/−^ and *Prm1*^−/−^ mice. Amplification of WT *Prm1* or the *Prm1*^−^ allele generated products of 437 bp or 270 bp, respectively. (C) IHC staining against PRM1 and PRM2 on Bouin-fixed, paraffin-embedded testis sections of *Prm1*^+/+^, *Prm1*^+/−^ and *Prm1*^−/−^ mice counterstained with Hematoxylin. (D) Mendelian distribution of genotypes (*n*=10 litters) from crossings of *Prm1*^+/−^ males and females. (E) Scatter plot of mean litter sizes monitored per male after mating with female WT C57BL/6J mice. The mean litter size per genotype is indicated by vermillion lines. (F) Pregnancy frequency (%) per male after mating with female WT C57BL/6J mice. *n*=number of males used; data are means and were analyzed using a two-tailed, unpaired Student's *t*-test (***P<0.001). Scale bars: 50 µm. L=ladder.

*Prm1*-deficient mice were generated using CRISPR-Cas9-mediated gene editing. Guide RNAs targeting exon 1 and exon 2 of *Prm1* and Cas9 mRNA were injected into zygotes. From the 13 pups obtained, four contained a deletion in the *Prm1*-coding region. Those four animals were mated to C57BL/6J mice and the *Prm1* locus was sequenced from the offspring. We selected a mouse carrying a 167 bp in-frame deletion in the *Prm1*-coding region (Fig. 1A) and established PCR-based genotyping (Fig. 1B).

In order to validate the deletion, 3′-mRNA-sequencing of whole testis of wild-type (WT; *Prm1*^+/+^) and *Prm1*^−/−^ males was performed. In *Prm1*^−/−^ males, the transcripts mapped to the 5′ and the 3′ ends of the *Prm1* locus, whereas we could not detect transcripts from the central deleted area, which encodes crucial arginine sites required for DNA binding (Fig. 1A; Fig. S1). In addition, read mapping to the surrounding *Prm2* and *Tp2* (*Tnp2*) loci was visualized to exclude local off-target effects, which would not be segregated via backcrossing (Fig. S2).

Next, we used immunohistochemical (IHC) staining with an anti-PRM1 antibody, targeting an epitope at the N terminus of PRM1 (marked in red in Fig. 1A) in order to determine whether the potential transcripts of the gene-edited allele resulted in the production of a truncated PRM1 protein. However, we could not detect a signal in testis sections of *Prm1*^−/−^ males (Fig. 1C). This strongly suggests nonsense-mediated RNA decay of the potential transcript and demonstrated that the deletion introduced by CRISPR-Cas9 resulted in a functional *Prm1*-null allele. PRM1 was detected in elongating spermatids and spermatozoa in WT and *Prm1*^+/−^ testis sections. PRM2 was present in all genotypes. Mating of *Prm1*^+/−^ males with *Prm1*^+/−^ females produced ∼50% *Prm1*^+/−^, ∼25% *Prm1*^+/+^ and ∼25% *Prm1*^−/−^ offspring (Fig. 1D), suggesting that the deletion did not interfere with embryonic development.

### *Prm1*^−/−^ male mice are infertile, while *Prm1*^+/−^ are subfertile

After establishing and validating the *Prm1*-deficient line, we performed fertility tests with *Prm1*^+/−^ and *Prm1*^−/−^ males. *Prm1*^+/−^ males were subfertile, whereas *Prm1*^−/−^ males were infertile (Fig. 1E; Table S1). None of the nine *Prm1*^−/−^ males tested was able to generate offspring. *Prm1*^+/−^ males generated smaller mean litter sizes (mean±s.d.: 2.88±2.20) compared with WT males (mean: 7.01±0.94) (Fig. 1E). Additionally, the pregnancy frequency of *Prm1*^+/−^ males was significantly reduced (Fig. 1F). Only ∼43% of the monitored copulations with *Prm1*^+/−^ males resulted in pregnancies. These results indicate that the loss of one allele of *Prm1* reduces male mouse fecundity.

### Spermatogenesis is overtly normal in *Prm1*-deficient mice

**Fig. 2.**
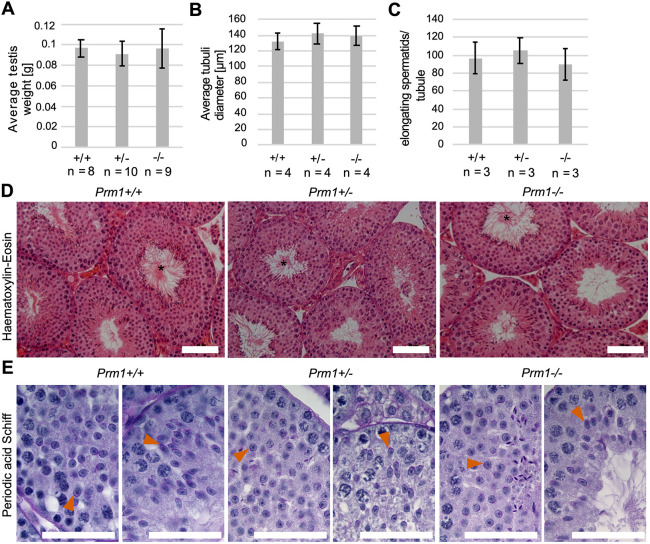
**Spermatogenesis of *Prm1*-deficient mice.** (A) Mean testis weight of *Prm1*^+/+^, *Prm1*^+/−^ and *Prm1*^−/−^ males (*n*=8-10). (B) Mean diameter of seminiferous tubules of *Prm1*^+/+^, *Prm1*^+/−^ and *Prm1*^−/−^ mice (*n*=4); 25 tubules per mouse were evaluated. (C) Quantification of elongating spermatids per seminiferous tubule cross-section in *Prm1*^+/+^, *Prm1*^+/−^ and *Prm1*^−/−^ males (*n*=3). Five tubules per mouse were evaluated. (D) Hematoxylin and Eosin staining of testis of *Prm1*^+/+^, *Prm1*^+/−^ and *Prm1*^−/−^ males. Tubules at stages VII to VIII of the epithelial cycle with spermatozoa lining up at the edge of tubule lumen are marked by asterisks. (E) PAS staining of testis of *Prm1*^+/+^, *Prm1*^+/−^ and *Prm1*^−/−^ males. Acrosomal structures are indicated by vermillion arrowheads. Data are mean±s.d. and were analyzed using a two-tailed, unpaired Student's *t*-test. Scale bars: 50 µm.

In order to test whether the deletion of *Prm1* affects spermatogenesis, we analyzed standard male fertility parameters. The mean testis weight (Fig. 2A), the mean seminiferous tubule diameter (Fig. 2B) and the number of elongating spermatids per seminiferous tubule cross-section (Fig. 2C) were not reduced in *Prm1*^+/−^ or *Prm1*^−/−^ animals compared with *Prm1*^+/+^ animals. Spermatozoa lining up at the lumen of stage VII to VIII seminiferous tubules were detected in *Prm1*-deficient mice (Fig. 2D). Spermatids underwent differentiation and elongation, and acrosomal structures and flagella were formed, in *Prm1-*deficient mice (Fig. 2E). Apparently, sperm development is overtly normal in *Prm1*^+/−^ and *Prm1*^−/−^ males.

### Epididymal *Prm1*-deficient sperm display ROS-mediated DNA damage

**Fig. 3.**
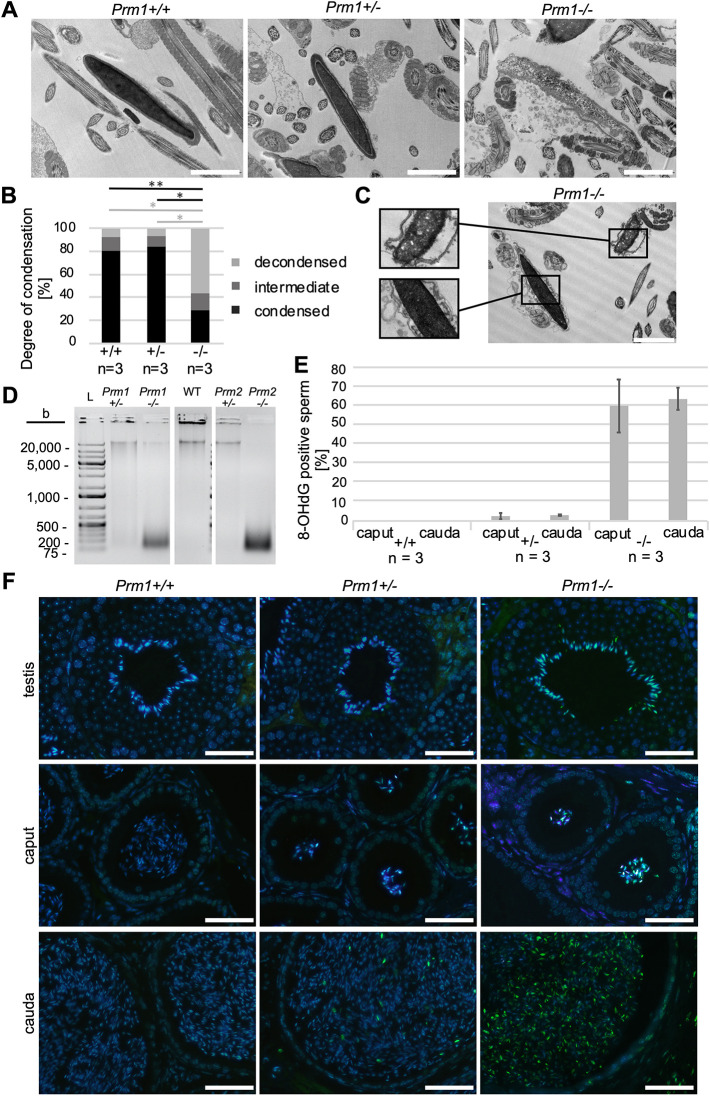
**Analysis of chromatin condensation and ROS-induced DNA damage in epididymal *Prm1*-deficient sperm.** (A) Representative transmission electron micrographs of *Prm1*^+/+^, *Prm1*^+/−^ and *Prm1*^−/−^ epididymal sperm. (B) Quantification of DNA condensation of epididymal sperm from *Prm1*^+/+^, *Prm1*^+/−^ and *Prm1*^−/−^ males (*n*=3); 100 sperm per male were analyzed. (C) Transmission electron micrograph of *Prm1*^−/−^ epididymal sperm. (D) Agarose gel loaded with genomic DNA isolated from epididymal sperm of *Prm1*^+/−^, *Prm1*^−/−^, *Prm2*^+/−^, *Prm2*^−/−^ and WT males separated by electrophoresis. Additional lanes loaded with ladders (L) were cut from the image. (E) Percentage of 8-OHdG-positive sperm on tissue sections of caput and cauda epididymis of *Prm1*^+/+^, *Prm1*^+/−^ and *Prm1*^−/−^ mice (*n*=3). (F) Representative IF staining against 8-OHdG in testis, caput epididymis and cauda epididymis tissue sections from *Prm1*^+/+^, *Prm1*^+/−^ and *Prm1*^−/−^ males. Data are mean±s.d. and were analyzed using a two-tailed, unpaired Student's *t*-test (*P<0.05; **P<0.005). Scale bars: 2 µm in A,C; 50 µm in F.

Given that PRM1 is necessary for DNA hypercondensation, we evaluated the chromatin compaction of epididymal sperm. Transmission electron micrographs of epididymal sperm revealed defects in chromatin hypercondensation in *Prm1*^−/−^ sperm compared with *Prm1*^+/−^ and *Prm1*^+/+^ sperm (Fig. 3A). Whereas ∼80-85% of *Prm1*^+/−^ and *Prm1*^+/+^ epididymal sperm nuclei appeared to be electron dense, indicative of condensed chromatin, only ∼29% of *Prm1*^−/−^ sperm nuclei appeared to be fully condensed (Fig. 3B). In addition, epididymal sperm from *Prm1*^−/−^ mice presented with membrane damage and disrupted acrosomes (Fig. 3C).

To assess DNA damage, genomic DNA isolated from epididymal sperm was separated by agarose gel electrophoresis. DNA from WT sperm presented as a single band of high molecular weight, indicative of intact DNA. By contrast, the majority of DNA isolated from *Prm1*^−/−^ epididymal sperm was detected as fragments of ∼100-500 bp, indicative of strong DNA degradation. Whereas DNA of sperm from *Prm2*^−/−^ male mice was completely fragmented, a small proportion of DNA in *Prm1*^−/−^ sperm presented as a high-molecular-weight band, indicating that a small portion of DNA from these sperm remained intact. DNA from *Prm1*^+/−^ sperm displayed a weak smear, indicative of a low, but detectable, level of DNA degradation (Fig. 3D). This suggests that the loss of one *Prm1* allele leads to low levels of DNA damage. This is in contrast to *Prm2*, where the loss of one allele was tolerated and DNA did not show any sign of degradation.

Given that similar DNA damage has been described for *Prm2*^−/−^ sperm and has been correlated to increased ROS levels during epididymal transit (Schneider et al., 2016, 2020), we stained testicular and epididymal tissue sections for 8-OHdG, a marker of oxidative stress-induced DNA lesions. In tissue sections from epididymides of *Prm1*^−/−^ mice, 60% of caput sperm and 64% of cauda sperm stained 8-OHdG-positive (Fig. 3E,F; Fig. S3). In epididymides of *Prm1*^+/−^ mice, a small number of sperm stained positive for 8-OHdG (mean: 2.6% in caput and 3.0% in cauda epididymis). In contrast, on sections of *Prm1*^+/+^ mice, no staining was detected. This shows that the low level of DNA damage detected in *Prm1*^+/−^ males is likely to be restricted to the few 8-OHdG-positive sperm and is not the result of a low level of DNA damage in all sperm. Of note, the majority of sperm from *Prm1*^−/−^ mice stained positive for 8-OHdG in the testis (Fig. 3F).

### Epididymal *Prm1*-deficient sperm display impaired membrane integrity, nuclear head morphology changes and sperm motility defects

**Fig. 4.**
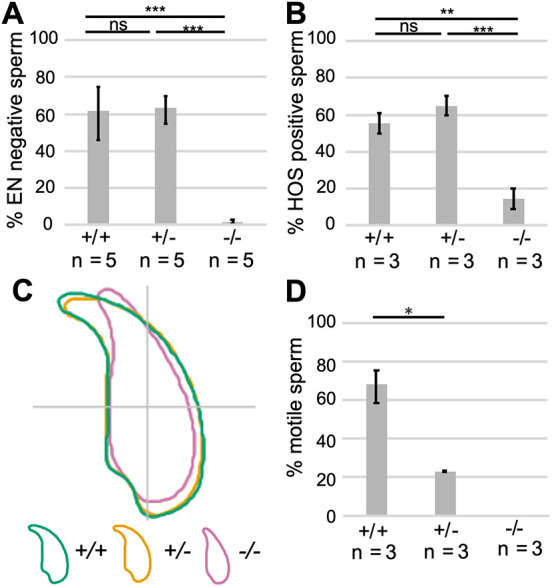
**Secondary effects on *Prm1*-deficient epididymal sperm.** (A) EN staining: quantification of EN-negative sperm (%) from *Prm1*^+/+^, *Prm1*^+/−^ and *Prm1*^−/−^ males (*n*=5). A minimum of 200 sperm per male were analyzed. (B) HOS test: quantification of HOS-positive sperm (%) from *Prm1*^+/+^, *Prm1*^+/−^ and *Prm1*^−/−^ males (*n*=3). A minimum of 200 sperm per male were analyzed. (C) Nuclear head morphology analysis for *Prm1*^+/+^, *Prm1*^+/−^ and *Prm1*^−/−^ sperm. Consensus shapes of sperm heads are depicted. Four males per genotype and a minimum of 100 sperm per animal were analyzed. (D) Quantification of motile and immotile sperm (%) from *Prm1*^+/+^, *Prm1*^+/−^ and *Prm1*^−/−^ males (*n*=3). Data are mean±s.d. and were analyzed using a two-tailed, unpaired Student's *t*-test (*P<0.05; **P<0.005; ***P<0.001). ns, not significant.

To characterize possible secondary effects of ROS, we next used Eosin-Nigrosin (EN) staining and a hypoosmotic swelling (HOS) test to examine sperm membrane integrity. *Prm1*^−/−^ epididymal sperm displayed severe membrane damage, indicative of inviable sperm, whereas no significant difference was detected between *Prm1*^+/−^ and *Prm1*^+/+^ sperm (Fig. 4A,B; Fig. S4).

To assess epididymal sperm head morphology, we used a high-throughput ImageJ plugin (Skinner et al., 2019) and generated a consensus shape visualizing the overall head shape of the population analyzed. *Prm1*^−/−^ sperm had lost the typical hook-shaped sperm head (Fig. 4C; Fig. S5A). Although the head shape of *Prm1*^−/−^ sperm displayed higher variability (Fig. S5B), they appeared to be smaller, with a mean area of 14.92 µm^2^ (95% CI 14.92±0.26) compared with 19.82 µm^2^ (95% CI 19.82±0.10) and 19.47 µm^2^ (95% CI 19.47±0.13) for *Prm1*^+/+^ and *Prm1*^+/−^ sperm heads, respectively (Fig. S5C). Furthermore, *Prm1*^−/−^ sperm heads were more elliptic (Fig. S5D) and thinner (Fig. S5E). *Prm1^+/−^* sperm heads showed a slightly stronger hook curvature, resulting in a reduced maximum Feret of 8.07 µm (95% CI 8.07±0.04) compared with 8.38 µm (95% CI 8.38±0.04) for *Prm1*^+/+^ sperm (Fig. S5F). Although the reduction in maximum Feret was significant, this result should be interpreted carefully, given the general variability but clear overlap in sperm head shapes depicted for *Prm1*^+/−^ and *Prm1*^+/+^ sperm populations (Fig. S5B). These results suggest that loss of one allele of *Prm1* does not affect sperm head shape dramatically. Periodic acid-Schiff stainings of seminiferous tubules showed that *Prm1*^−/−^ sperm form a hook during spermiogenesis (Fig. S6A). The consensus sperm head shapes generated using step 14-16 spermatozoa isolated from testis did not differ overtly among the different genotypes (Fig. S6B), indicating that *Prm1*^−/−^ sperm lose their typical hooked head shape during epididymal transit.

**Fig. 5.**
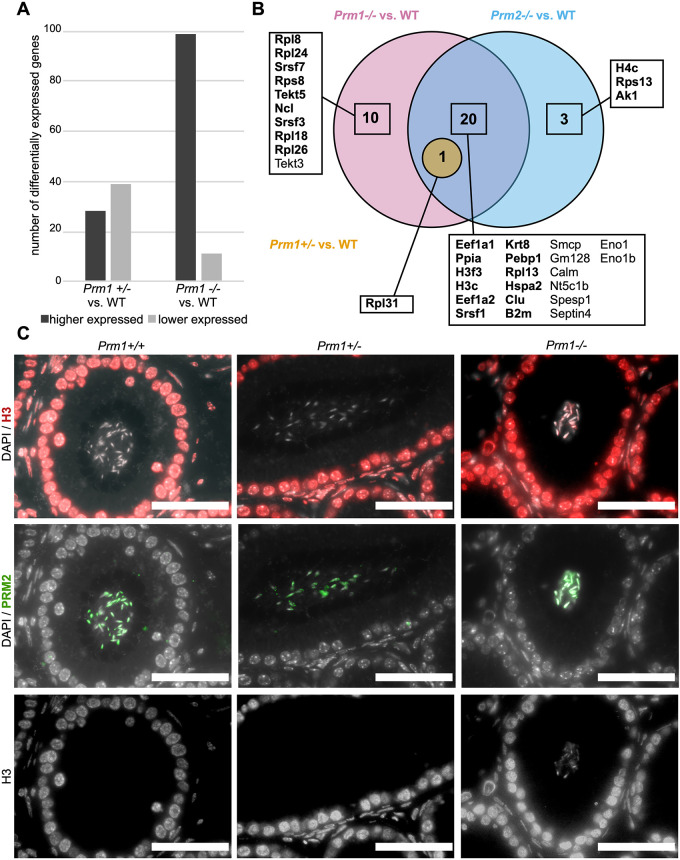
**Differentially expressed genes in the testis and altered protein abundances in sperm in protamine-deficient males.** (A) Number of differentially expressed genes subdivided into higher and lower expressed genes in testis of *Prm1*^+/−^ and *Prm1*^−/−^ males compared with WT males. (B) Venn diagram illustrating changes in abundances of proteins from sperm basic protein extractions of *Prm1*^−/−^, *Prm1*^+/−^ and *Prm2*^−/−^ males compared with WT. Proteins that were more abundant are in bold. Non-bold proteins showed lower abundance compared with WT. (C) IHC stainings against histone H3 (red) and PRM2 (green) of *Prm1*^+/+^, *Prm1*^+/−^ and *Prm1*^−/−^ caput epididymal tissue sections. DAPI (in gray) was used as the counterstain. The H3 stainings are additionally shown as single gray channel pictures. Scale bars: 50 µm.

Next, we analyzed the percentage of motile sperm isolated from the cauda epididymis (Fig. 5D). Strikingly, *Prm1*^+/−^ sperm showed a marked reduction in sperm motility. Only around 23% of *Prm1*^+/−^ sperm were motile. In contrast, 68% of WT sperm were motile, while *Prm1*-deficient sperm were completely immotile. We would like to point out that sperm from the cauda epididymis were isolated using the swim-out assay, which could potentially bias the overall motility assessment toward motile sperm. The percentage of motile sperm in the *Prm1*^+/+^ samples was, however, in the range of published data (Goodson et al., 2011), *Prm1*^+/−^ sperm exhibited significantly lower motility. Thus, the reduction in motility is likely to contribute to the sub/infertility seen in these sperm. Transmission electron micrographs of flagella cross-sections of epididymal sperm showed that a large part of the *Prm1*^+/−^ and *Prm1*^−/−^ sperm did not have the characteristic ‘9+2’ microtubule structure (Fig. S7A-C). While 98% of *Prm1*^+/+^ sperm flagella showed a ‘9+2’ microtubule structure, this was seen in only 69% of *Prm1*^+/−^ and 54% of *Prm1*^−/−^ sperm flagella (Fig. S7D). Furthermore, severe flagellar membrane damage was seen in *Prm1*^−/−^ sperm tails. In transmission electron micrographs of *Prm1*^−/−^ seminiferous tubules, flagella formation and sperm head shaping appeared to be normal, suggesting that sperm tail damage accumulates during epididymal transit (Fig. S8). Noteworthy, sperm chromatin appeared to be electron dense in the testis, indicative of condensed DNA, again suggesting that severe DNA damage and fragmentation accumulate after spermiation.

### Transcriptional and proteomic profiling reveals differences in *Prm1* and *Prm2*-deficient male mice

To determine whether transcription is affected upon loss of protamines, we performed transcriptomic and proteomic analyses. Whole testis 3′-mRNA-sequencing revealed that, compared with WT testis, 99 genes showed higher and 11 showed lower expression in *Prm1*^−/−^ testis, whereas 28 genes showed higher and 39 showed lower expression in *Prm1*^+/−^ testis (Fig. 5A; Table S2). In *Prm1*^−/−^ testis, pathway enrichment for immune-related genes (*Il1b*, *Ccl5*, *Saa3*, *Atp6ap1*, *Rsad2*, *Cxcl10*, *Ifit1*, *Mmp13*, *Clec4e* and *Zdhhc1*) was identified. These transcripts were slightly more abundant in *Prm1*^−/−^ testis compared with WT testis, but showed overall low levels of expression (Table S2). This might indicate a reaction to ROS-induced damage or slight contamination with blood cells.

In order to determine whether proteins might be differentially abundant in mature sperm, we used mass spectrometry to analyze basic nuclear protein extracts of *Prm1*^+/−^, *Prm1*^−/−^, *Prm2*^−/−^ and WT sperm. In *Prm1*^−/−^ samples, 31 proteins were differentially abundant compared with WT sperm (Fig. 5B, Table S3). Of these, 21 were also differentially abundant in *Prm2*^−/−^ samples. Proteins related to translation, mRNA splicing and protein folding (EEF1A1, EEF1A2, RPL13, RPL31, SRSF1 and PPIA) showed higher abundance in *Prm1*^−/−^ or *Prm2*^−/−^ sperm compared with WT sperm. Additionally, histones (H3F3, H3C) were more abundant in *Prm1*^−/−^ and *Prm2*^−/−^ sperm, indicating increased H3 histone retention. In addition, in *Prm2^−/−^* sperm histone H4C was also more abundant. Increased histone retention was validated using immunofluorescence (IF) staining against H3 on caput epididymal tissue sections (Fig. 5C; Fig. S9). H3 was detected in sperm on *Prm1*^−/−^ and *Prm2*^−/−^ sections, but not on *Prm1*^+/−^, *Prm2*^+/−^ or WT sections. H4 was detected in *Prm2*^−/−^ sperm, but not in *Prm1*^+/−^, *Prm2*^+/−^ or WT sperm. Additionally, weak staining of H4 was detected in *Prm1*^−/−^ sperm.

In *Prm1*^−/−^ samples, further proteins were detected to be more abundant related to translation and mRNA splicing (RPL8, RPS8, RPL18, RPL24, RPL26, SRSF3 and SRSF7). Proteins related to stress response and apoptosis (B2M and CLU) were also more abundant in sperm lacking PRM1 or PRM2. This might reflect a stress response to the increased ROS-mediated sperm damage detected. By contrast, SMCP and SPESP1, proteins important for sperm motility and sperm-egg fusion, were less abundant in both *Prm1*^−/−^ and *Prm2*^−/−^ samples. Interestingly, heat shock-related 70 kDa protein 2 (HSPA2), which was more abundant in both *Prm1*^−/−^ and *Prm2*^−/−^ samples, has been proposed to function as a transition protein (TP) chaperone in condensing spermatids (Govin et al., 2006). In order to test whether an elevated level of HSPA2 affects the presence of TPs, we used IF staining against TP1 (TNP1) and TP2 (TNP2) on *Prm1*^−/−^, *Prm1*^+/−^ and *Prm1*^+/+^ caput epididymis tissue sections (Fig. S10). TP1 and TP2 were detected in caput *Prm1*^−/−^ sperm, but not in *Prm1*^+/−^ or *Prm1*^+/+^ sections. A higher abundance of HSPA2 in *Prm1*^−/−^ and *Prm2*^−/−^ epidydimal sperm compared with WT could be indicative of TP-HSPA2 complexes and, in turn, impaired or incomplete TP unloading. As shown by co-IF staining against TP1 and PRM2, the majority of TP1-positive *Prm1*^−/−^ caput sperm stained positive for both proteins.

Only one protein, the ribosomal protein RPL31, which was also identified in *Prm1*^−/−^ and *Prm2*^−/−^ samples, was more abundant in *Prm1*^+/−^ sperm basic protein extracts compared with WT sperm. The fact that there was only one non-protamine protein differentially abundant in *Prm1*^+/−^ basic protein extracts suggests that the *Prm1*^+/−^ sperm protein profile is not causative of the subfertility observed in these mice.

### Protamine and basic protein content are altered in protamine-deficient epididymal sperm

**Fig. 6.**
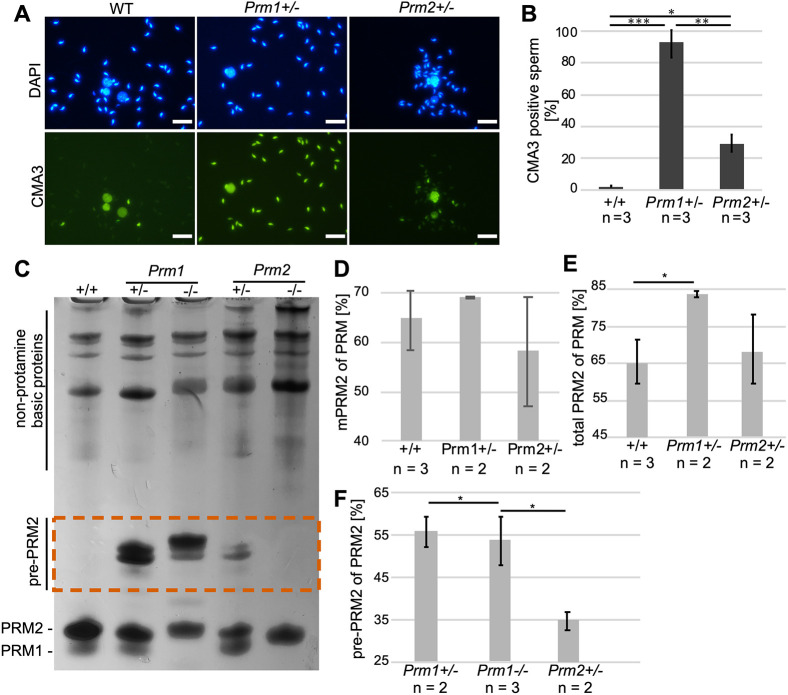
**Sperm basic protein analysis in protamine-deficient sperm.** (A) Representative images of CMA3 staining of *Prm1*^+/+^, *Prm1*^+/−^ and *Prm2*^+/−^ epididymal sperm heads taken at the same exposure time. DAPI was used as a counterstain. (B) Mean percentage of CMA3-positive sperm in *Prm1*^+/−^, *Prm2*^+/−^ and WT males (*n*=3). A minimum of 400 sperm per male were analyzed. (C) Representative AU-PAGE of basic protein extractions from WT, *Prm1*^+/−^, *Prm1*^−/−^, *Prm2*^+/−^ and *Prm2*^−/−^ epididymal sperm. Non-protamine basic proteins were detected at the top of the AU-PAGE. PRM1 and PRM2 ran at the bottom of the gel. Pre-PRM2 ran higher than did mature PRM (dashed vermillion box). (D) Percentage of mPRM2 of PRM in basic protein extractions from WT, *Prm1*^+/−^ and *Prm2*^+/−^ epididymal sperm. (E) Percentage of total PRM2 (including pre-PRM2) of PRM in basic protein extractions from WT, *Prm1*^+/−^ and *Prm2*^+/−^ epididymal sperm. (F) Percentage of pre-PRM2 of PRM2 in basic protein extractions from *Prm1*^+/−^, *Prm1*^−/−^ and *Prm2*^+/−^ epididymal sperm. Data are mean±s.d. and were analyzed using a two-tailed, unpaired Student's *t*-test (*P<0.05; **P<0.005; ***P<0.001). Scale bars: 20 µm.

Next, we analyzed the level of protamination using Chromomycin A3 (CMA3), a dye competing with protamines to bind CG-rich regions to the minor groove of DNA (Sadeghi et al., 2019). Whereas 98% of *Prm1*^+/−^ sperm showed CMA3 staining, only ∼29% of *Prm2*^+/−^ sperm showed a CMA3 signal (Fig. 6A,B). These data suggest that chromatin in *Prm1*^+/−^ and *Prm2*^+/−^ sperm is either not fully or not correctly protaminated, with the effects being more dramatic in *Prm1*^+/−^ sperm. Of note, sperm from *Prm1*^−/−^ and *Prm2*^−/−^ mice could not be analyzed because severe DNA fragmentation interfered with the staining procedure.

To further analyze the relative protamine content and protamination of epididymal sperm of *Prm1*^+/−^, *Prm1*^−/−^, *Prm2*^+/−^, Prm2^−/−^ and WT mice in more detail, basic proteins were separated on acid-urea polyacrylamide gels (AU-PAGE). Most interestingly, in sperm from *Prm1*^+/−^ and *Prm1*^−/−^ mice, PRM2 precursors (pre-PRM2) were detected, suggesting disturbances in processing of PRM2 upon loss of PRM1 (Fig. 6C, dashed vermillion box). Furthermore, we quantified the relative amounts of basic proteins within individual samples (Fig. 6D-G; Fig. S11). Of note, basic protein extractions are enriched for nuclear proteins, but also contain other basic sperm proteins. We identified ODF2, GPX4 and SPAG8 to contribute to the prominent Coomassie-stained bands in the upper part of the AU-PAGE (Fig. S12A). Noteworthy, canonical histones or testis-specific histone variants ran mainly in the middle of the gel (Fig. S12A,B).

While the relative amount of mPRM2 to total protamine was not significantly different in *Prm1*^+/−^ and *Prm2*^+/−^ sperm compared with WT (Fig. 6D), the total amount of PRM2 (mPRM2+pre-PRM2) was significantly higher in *Prm1*^+/−^ sperm (83%) only (Fig. 6E). Taking these data into account, the PRM1:PRM2 ratio in *Prm1*^+/−^ sperm shifted to ∼1:5, whereas the species-specific protamine ratio of 1:2 was maintained in *Prm2*^+/−^ sperm, which is comparable to WT (Corzett et al., 2002). Consequently, the pre-PRM2 content of total PRM2 was significantly larger in *Prm1*^+/−^ and *Prm1*^−/−^ sperm compared with *Prm2*^+/−^ sperm (Fig. 6F).

## DISCUSSION

In this study, mice deficient for *Prm1* were generated using CRISPR-Cas9-mediated gene editing. *Prm1*^−/−^ male mice were infertile, whereas loss of one allele of *Prm1* resulted in subfertility. *Prm1*^−/−^ sperm showed severe DNA fragmentation, high levels of 8-OHdG, destructured membranes and complete immotility. *Prm1*^+/−^ sperm showed moderate ROS-induced DNA damage, reduced sperm motility and a shifted PRM1:PRM2 ratio. *Prm1*^−/−^ and *Prm1*^+/−^ sperm contained high levels of incompletely processed PRM2, suggesting that PRM1 is necessary for correct PRM2 processing.

Protamine-deficient mouse models have been described and associated with male factor infertility in previous studies (Cho et al., 2003, 2001; Takeda et al., 2016; Mashiko et al., 2013). However, contrary to previous studies, we show that *Prm1*^+/−^ males were able to produce offspring by natural breeding. *Prm1*-deficient chimeras, which have been generated by classical gene-targeting techniques, were reported to be infertile (Cho et al., 2001), excluding mouse line establishment and detailed studies on *Prm1* deficiency. Takeda et al. were, however, able to generate viable offspring from *Prm1*^+/−^ males by *in vitro* fertilization (IVF) of zona-free oocytes (Takeda et al., 2016). Furthermore, Mashiko et al. reported that CRISPR-Cas9-mediated *Prm1*^+/−^ mice were infertile, although detailed fertility statistics and phenotypical analysis of *Prm1*-deficient mice were not performed (Mashiko et al., 2013). Given that the *Prm1*^+/−^ males in the current study were subfertile, we were able to generate and analyze *Prm1*^−/−^ mice. Takeda et al. used embryonic stem cell (ESC)-targeting technology, which might explain the differences in *Prm1*^+/−^ fertility. In contrast, Mashiko et al. used both the identical strain (C57BL/6J × DBA, backcrossed to C57BL/6J) and technology to the current study, they might not have performed a sufficiently exhaustive fertility analysis in order to detect subfertility.

Spermatogenesis was overtly normal in *Prm1*^−/−^ (and *Prm1*^+/−^) mice compared with WT mice. Similar results were described for *Prm2*^−/−^ mice, in which spermatogenesis appears to be orderly (Schneider et al., 2016) whereas epididymal sperm show severe damage. It was reported that an oxidative stress-mediated destruction cascade is initiated during epididymal sperm maturation in *Prm2*^−/−^ mice (Schneider et al., 2016, 2020). While it is well known that low levels of ROS are required for proper sperm function, high levels cause sperm pathologies (de Lamirande et al., 1997). Accumulation of ROS and loss of the antioxidant capacity of *Prm2*^−/−^ sperm caused severe DNA fragmentation, sperm immotility and sperm membrane damage. We have observed even earlier effects in *Prm1*^−/−^ mice displaying ROS-mediated DNA damage in the testis already (Schneider et al., 2016). Thus, loss of *Prm1* renders the ROS system more fragile at an even earlier stage of sperm development. Of note, abnormal epididymal sperm head shapes were detected for both *Prm1*^−/−^ and *Prm2*^−/−^ sperm (Schneider et al., 2020). However, sperm isolated from the testis showed normal head shapes, suggesting that the altered head shapes are caused by increasing levels of ROS.

Transcriptional profiling of the whole testis revealed only small differences in gene expression in *Prm1*^−/−^ males compared with *Prm1*^+/−^ and *Prm1*^+/+^ males. However, we did detect increased histone retention in *Prm1*^−/−^ and *Prm2*^−/−^ sperm using mass spectrometry and IF. Differential abundance analysis of basic proteins in *Prm1*^−/−^ and *Prm2*^−/−^ epididymal sperm showed proteins related to translation and apoptotic processes, consistent with the secondary effects observed. However, only moderate differences were detected in *Prm1*^+/−^ sperm compared with WT, indicating that these changes are unlikely to contribute to the phenotype observed. Interestingly, HSPA2, which was proposed as a TP chaperone (Govin et al., 2006), was more abundant in *Prm1*^−/−^ and *Prm2*^−/−^ sperm compared with WT. Given that we detected slight transition protein retention in *Prm1*^−/−^, but not in *Prm1*^+/−^ caput epidydimal sperm, this might indicate that TP unloading is disturbed in *Prm1*^−/−^ sperm, leading to the more abundant HSPA2 protein level.

Whereas *Prm1*^−/−^ male mice displayed a phenocopy of *Prm2*^−/−^ male mice, marked differences were found between heterozygous males. Interestingly, *Prm1*^+/−^ males were subfertile, showing a reduction in mean litter sizes and lower pregnancy frequencies. Of note, *Prm2*^+/−^ are fertile (Schneider et al., 2016). This suggests that the loss of one allele of *Prm1*, in contrast to the loss of one allele of *Prm2*, cannot be tolerated. Transmission electron micrographs revealed that DNA of *Prm1*^+/−^ sperm appeared to be electron dense, suggesting that the chromatin in sperm is condensed to the same level as in *Prm2*^+/−^ (Schneider et al., 2016) and WT sperm. This raises the question as to why *Prm1*^+/−^ males are subfertile.

We showed that a small population of *Prm1*^+/−^ epididymal sperm stained positive for 8-OHdG. In addition, genomic DNA isolated from *Prm1*^+/−^ sperm was partially fragmented. This indicates that some sperm experience DNA damage caused by ROS rather than all sperm bearing some degree of DNA damage. Surprisingly, however, we did not detect marked differences in chromatin condensation or membrane integrity between WT and *Prm1*^+/−^ sperm. *Prm2*^+/−^ sperm do not show an increase in ROS-mediated DNA damage compared with WT sperm (Schneider et al., 2020). Thus, *Prm1*^+/−^ sperm appear to be more sensitive or more exposed to oxidative stress-mediated damage compared with *Prm2*^+/−^ sperm. This might contribute to the subfertility of *Prm1*^+/−^ males.

Noteworthy, redox imbalance in sperm has been repeatedly connected to not only sperm DNA damage, but also reduced sperm motility in men (Alahmar, 2019). It has been reported that sperm mitochondria present a significant source of ROS in defective sperm (Koppers et al., 2008). In humans, spontaneous production of mitochondrial ROS by defective sperm causes peroxidative damage to the sperm midpiece, leading to reduced sperm motility. One of the major differences between *Prm1*^+/−^ and *Prm2*^+/−^ sperm is that the loss of one allele of *Prm1* leads to a marked decrease in sperm motility, whereas *Prm2*^+/−^ sperm motility is not significantly different from that of WT sperm (Schneider et al., 2016). Only ∼23% of *Prm1*^+/−^ sperm were motile, an amount that, in humans, qualifies as asthenozoospermic, according to the WHO criteria (Alahmar, 2019; Cooper et al., 2010; World Health Organization, 2010). Given that mitochondrial ROS is negatively correlated with sperm motility and we detected a moderate increase in ROS in *Prm1*^+/−^ sperm compared with WT, we believe that reduced sperm motility in *Prm1*^+/−^ males is (at least partially) caused by ROS, which contributes to the subfertility observed in *Prm1*^+/−^ males. Of note, motility defects were described in *Prm1*^+/−^ mice generated by Takeda et al. (2016). They reported that *Prm1*^+/−^ sperm show a reduction in the mitochondrial membrane potential, which has been associated with reduced sperm motility (Gawlik et al., 2008). Furthermore, scanning electron microscopy revealed various tail deformities in *Prm1*^+/−^ sperm. Here, we found similar sperm tail defects in transmission electron micrographs of *Prm1*^+/−^ and *Prm1*^−/−^ sperm.

Another notable difference between *Prm1*^+/−^ and *Prm2*^+/−^ sperm was the aberrant DNA protamination revealed by CMA3 staining. While approximately one-third of *Prm2*^+/−^ sperm stained with CMA3, ∼98% of *Prm1*^+/−^ sperm were CMA3 positive. In human ejaculates, the percentage of CMA3-positive sperm varies considerably (Lolis et al., 1996) and values of up to 30% CMA3-positive sperm have been defined for normal semen samples from fertile men (Sakkas et al., 1996; Zandemami et al., 2012). Thus, we argue that the 29% of CMA3-positive sperm seen in *Prm2*^+/−^ males, despite being higher than the values detected in WT controls, can be tolerated and do not affect regular fertility. However, it is surprising that only 2% of CMA3-negative sperm in *Prm1*^+/−^ mice still resulted in a partially retained fertility. Enhanced CMA3 staining of sperm was correlated with increased histone retention. Surprisingly, the high CMA3 level in *Prm1*^+/−^ sperm could not be correlated with increased histone retention as shown by mass spectrometry and IF. One possible explanation for the intense CMA3 staining in *Prm1*^+/−^ sperm could be the vast amounts of pre-PRM2 detected in basic protein extracts from epididymal sperm. We hypothesize that the failure of processing of pre-PRM2 and pre-PRM2 loading onto sperm DNA might allow the intercalating dye to access DNA and stain the chromatin.

Defects in PRM2 processing were also described for *Prm1*^+/−^ mice developed by Takeda et al., as well as in histone variant H2A.L.2-knockout (KO), TP1/TP2-double KO, TP2-KO and cleaved PRM2 cP2-KO mouse models (Arévalo et al., 2022; Zhao et al., 2001; Barral et al., 2017; Shirley et al., 2004; Takeda et al., 2016), all of which display fertility problems. Interestingly, a recent study showed that mutation of a single non-arginine residue in PRM1 (P1^K49A/K49A^) leads to impaired PRM2 processing in mice (Moritz et al., 2021 preprint). Of note, *Prm2*^+/−^ sperm also contain scarce amounts of pre-PRM2. However, the relative amount of pre-PRM2 is significantly larger in *Prm1*^+/−^ sperm. Hence, species-specific PRM1 levels are required for accurate PRM2 processing and alterations of these levels unequivocally lead to reduced fertility. Noteworthy, the presence of pre-PRM2 in subfertile human sperm has been described previously (de Yebra et al., 1998).

In addition to high levels of pre-PRM2, *Prm1*^+/−^ sperm displayed a shift in the PRM1:PRM2 ratio from ∼1:2 in WT sperm to 1:5 in *Prm1*^+/−^ sperm. Furthermore, in cP2-deficient mice, a shift in the protamine ratio has been described. Arévalo et al. have shown that mice lacking the highly conserved N-terminal part of PRM2, called cleaved PRM2 (cP2), display defective PRM2 processing and a PRM1:PRM2 ratio of ∼5:1 (Arévalo et al., 2022). Mice lacking cP2 on one allele are infertile. Given that *Prm1*^+/−^ males were subfertile, it appears that a ratio of 1:5 can be tolerated to some extent, whereas a 5:1 ratio is incompatible with fertility. Of note, the protamine ratio in *Prm2*^+/−^ sperm was not significantly different from WT sperm, explaining their regular fertility. In humans, alterations of the species-specific ratio at both the protein and transcript level have been repeatedly correlated with male sub- and infertility (Aoki et al., 2005; Balhorn et al., 1988; Belokopytova et al., 1993; Bench et al., 1998; de Yebra et al., 1998; García-Peiró et al., 2011; Khara et al., 1997; Ni et al., 2016; Oliva, 2006; Steger et al., 2001, 2003, 2008; Torregrosa et al., 2006). These results once again underline the importance of the protamine ratio in species expressing both protamines.

Of note, the protamine ratio in mice harboring a C-terminally altered allele of protamine 1 (P1^K49A/K49A^) was unaltered, contrary to the *Prm1*^+/−^ sperm analyzed here (Moritz et al., 2021 preprint). Similar to *Prm1*^+/−^ mice, P1^K49A/K49A^ mice are subfertile. Interestingly, P1^K49A/K49A^ sperm show increased histone retention, similar to *Prm1*^−/−^ and *Prm2*^−/−^ sperm, which was not detected in *Prm1* ^+/−^ sperm. Thus, the presence of one functional *Prm1* allele is sufficient for accurate histone eviction.

In summary, we generated and characterized *Prm1*-deficient mice. We demonstrated that *Prm1*^+/−^ mice are subfertile, exhibiting sperm with moderate ROS-induced DNA damage and reduced motility. Compared with *Prm2*^+/−^ sperm, large amounts of pre-PRM2 were detected in *Prm1*^+/−^ sperm. Although the crucial species-specific protamine ratio was maintained in *Prm2*^+/−^ sperm, *Prm1*^+/−^ sperm exhibited an aberrant protamine ratio. We also demonstrated that *Prm1*^−/−^ and *Prm2*^−/−^ mice displayed increased histone retention and redox imbalance, leading to severe sperm damage, which rendered males infertile. Loss of *Prm1* appeared to trigger the ROS system at an even earlier stage compared with the loss of *Prm2*. By intercrossing the *Prm1*-deficient mouse line presented here with our previously published *Prm2*^+/−^ model, we will next generate and analyze *Prm1*^+/−^ *Prm2*^+/−^ double-heterozygous males, which will further advance our knowledge of the molecular consequences of disturbances in PRM1 and PRM2 levels and the PRM1:PRM2 ratio.

## MATERIALS AND METHODS

### Ethics statement

All animal experiments were conducted according to the German law of animal protection and in agreement with the approval of the local institutional animal care committees (Landesamt für Natur, Umwelt und Verbraucherschutz, North Rhine-Westphalia, approval ID: AZ84-02.04.2013.A429; AZ81-0204.2018.A369).

### Generation of *Prm1*-deficient mice

Single guide (sg)RNAs (sg1_ts: 5′-CACCGCGAAGATGTCGCAGACGG; sg1_bs: 5′-AAACCCGTCTGCGACATCTTCGC; sg2_ts: 5′-CACCGTGTATGAGCGGCGGCGA, sg2_bs: 5′-AAACTCGCCGCCGCTCATACAC) were tested in ESCs as described previously (Schneider et al., 2016). The sgRNAs targeted exon 1 and exon 2 of *Prm1*.

CRISPR-Cas9-mediated gene editing of zygotes was performed as described previously (Schneider et al., 2016). In brief, 6-8-week-old B6D2F1 females were superovulated by intraperitoneal injections of 5 i.u. pregnant mare's serum (PMS; hor-272-b, ProSpec) and 5 i.u. human chorionic gonadotropin (hCG; Orogest, MSD). Females were mated with B6D2F1 males and zygotes were isolated 0.5 days post copulation (dpc). sgRNAs (50 ng/µl each) were microinjected together with Cas9 mRNA (100 ng/µl). After culturing in KSOM medium (G-TL; 10145, Vitrolife) for 3 days, developing blastocysts were transferred into the uteri of pseudo-pregnant CB6F1 foster mice. Offspring were genotyped by PCR and sequenced to identify founder animals. After first backcrossing to C57BL/6J mice, the F1 generation was sequenced. The allele (NM_013637.5:c.51_125del) was further back-crossed to C57BL/6J mice. Starting from the N4 generation, analyses were performed using male mice aged between 8 and 13 weeks.

### *Prm2*-deficient mice

*Prm2*-deficient mice (MGI: 5760133; 5770554) generated and analyzed by Schneider et al. (2016, 2020) were used for comparison with *Prm1*-deficient mice and WT.

### Genotyping and sequencing of mice

Primers flanking the gene-edited region (Prm1_fwd: 5′-CCACAGCCCACAAAATTCCAC, Prm1_rev: 5′-TCGGACGGTGGCATTTTTCA) were used to amplify both the WT and edited alleles [cycling conditions: 2 min 95°C; 30× (30 s 95°C; 30 s 64°C; 35 s 72°C); 5 min 72°C]. PCR products (WT allele, 437 bp; *Prm1Δ*167, 270 bp) were separated on agarose gels.

PCR products were cloned using the TOPO TA Cloning Kit with pCR 2.1-TOPO (Thermo Fisher Scientific) according to the manufacturer's instructions. Plasmids were transformed into E.cloni 10G Chemically Competent Cells (Lucigen) according to the manufacturer's instructions, isolated by alkaline lysis and sequenced by GATC/Eurofins.

### Fertility assessment

Fertility was tested by mating male mice 1:1/1:2 to C57BL/6J females. Females were examined for the presence of a vaginal plug daily. Plug-positive females were separated and monitored. Pregnancies and litter sizes were recorded. A minimum of five plugs per male were evaluated. Male mice entered the fertility testing aged between 8 and 13 weeks. Female mice were aged between 10 and 16 weeks.

### Immunohistochemistry/immunofluorescence

Tissues were fixed in Bouin's solution (75% saturated picric acid, 10% formalin, 5% glacial acetic acid) or paraformaldehyde (PFA; 4% w/v PFA/water) (4°C, overnight) and processed in paraffin; 3 µm sections were then generated using a microtome (Microm CP60). After deparaffinization, slides were treated with decondensation buffer, as described previously (Schneider et al., 2020). Heat-mediated antigen retrieval was performed (citrate buffer pH 6.0) for 20 min, followed by blocking in Tris-HCl buffer (pH 7.4, 5% bovine serum albumin, 0.5% Triton X-100) and primary antibody treatment overnight at 4°C. For IHC staining against protamines [anti-PRM1 (Hup1N) and anti-PRM2 (Hup2B) Briar Patch Biosciences; 1:200], slides were treated with 3% H_2_O_2_ for 30 min after decondensation. Biotinylated goat-anti-mouse antibodies (Dako; E0433; 1:200) was used as secondary antibodies (1 h, room temperature), processed using a Vectastain Elite ABC-HRP Kit (Vector Laboratories; PK-6100) and stained with AEC-solution (Dako, AEC+ Substrate, K3469). Counterstain was performed using Hematoxylin. For IF against 8-OHdG (Santa Cruz Biotechnology; sc-66036; 1:200), goat-anti-mouse Alexa Fluor 488 antibodies (Thermo Fisher Scientific; A-11001; 1:500) were used as secondary antibodies for 2 h at room temperature. Nuclei were stained using 1 µg/ml Hoechst (Thermo Fisher Scientific; 33342). 8-OHdG-positive sperm were quantified using the Adobe Photoshop counting tool. Two tubule cross-sections per organ per mouse for three animals per genotype were analyzed. Slides for IF against TP1 (Abcam; ab73135; 1:1000), TNP2 (Santa Cruz Biotechnology; sc-393843; 1:100), H3 (Abcam; ab1791; 1:1500), H4 (Abcam; ab177840; 1:2000) and PRM2 (Briar Patch Biosciences; 1:200) were processed using the VectaFluor Duet Double Labeling Kit (Vector Laboratories; DK-8828) and mounted with ROTI Mount FluorCare DAPI (Carl Roth).

### Macroscopic analysis of testis

Sections of Bouin-fixed testis were deparaffinized, hydrated, stained with Hemalum solution acid (Henricks and Mayer, 1965) and Eosin Y solution (Carl Roth), dehydrated and mounted with Entellan (Sigma-Aldrich/Merck). Tubule diameters were determined by measuring the horizontal and vertical diameters of at least 25 tubules per testis cross-section. The number of elongated spermatids per tubules for a minimum of five tubules per mouse was counted with the ImageJ cell counter. Images were white balanced using the ImageJ macro ‘White balance correction_1.0’ originally written by Vytas Bindokas (2006, University of Chicago, USA) and modified by Patrice Mascalchi (2014, University of Cambridge, UK).

### Periodic Acid Schiff staining

Periodic Acid Schiff (PAS) staining was performed as previously described (Schneider et al., 2020). After deparaffinization and rehydration, slides were incubated for 10 min in periodic acid (0.5%), rinsed in H_2_O, incubated for 20 min with Schiff reagent, counterstained and coverslipped.

### Isolation of epididymal sperm

Sperm were isolated from the cauda epididymis by swim-out as previously described (Schneider et al., 2016). The epididymal tissue was incised multiple times and incubated in M2 medium (Sigma-Aldrich) or PBS at 37°C for 15-30 min.

### Transmission electron microscopy

Isolated sperm were pelleted (10,000 ***g***, 2 min), fixed in 3% glutaraldehyde at 4°C overnight, washed with 0.1 M cacodylate buffer (twice for 15 min each), post-fixed with 2% osmium tetroxide at 4°C for 2 h and washed again. After dehydration in an ascending ethanol series and contrasting in 70% (v/v) ethanol 0.5% (m/v) uranyl acetate (1-1.5 h, 4°C), samples were washed with propylenoxide (three times for 10 min each at room temperature) and stored in propylenoxide:Epon C [1:1 (v/v)] at 4°C overnight. Next, the pellets were embedded in Epon C (70°C, 48 h). Ultra-thin sections were examined using a Philips CM10 transmission electron microscope equipped with analySiS imaging software. Using ImageJ, 100 sperm per sample were analyzed to determine the difference between the minimum and maximum gray values. Chromatin condensation status was categorized according to high (<150), intermediate (150-180) and low (>180) differences in gray scale. Sections used to examine sperm flagella were taken with a scanning electron microscope (FEI Verios 460L) equipped with a STEM3 detector.

### Assessment of sperm DNA integrity

Sperm genomic DNA was isolated as previously described (Weyrich, 2012) with minor adjustments. Briefly, sperm were incubated in 500 µl lysis buffer [1 M Tris-HCl pH 8.0, 3 M NaCl, 0.5 M EDTA, 20% (m/v) SDS] supplemented with 21 µl 1 M DTT, 2.5 µl 0.5% Triton X-100 and 40 µl 10 mg/ml proteinase K at 50°C overnight. After centrifugation (15,500 ***g***, 10 min), 1 µl 20 mg/ml glycogen and 1/10 vol 3 M NaAc were added to the supernatant. Precipitation was performed using absolute ethanol for 2 h at −80°C followed by 45 min at −20°C. The pellet was washed with 75% ethanol and dried in a Speed Vac DNA110 (Savant). DNA was dissolved in 30 µl TE buffer (10 mM Tris/HCl, 1 mM EDTA, pH 8.0).

### Chromomycin A3 staining

Epididymal sperm were fixed in Carnoy’s solution [3:1 methanol:acetic acid (v/v)], spread on microscope slides and covered with 100 µl CMA3 solution [0.25 mg/ml CMA3 in Mcllvaine buffer (pH 7.0, containing 10 mM MgCl_2_)]. After incubation for 20 min in the dark, slides were rinsed with Mcllvaine buffer and mounted with ROTI Mount FluorCare DAPI. In total, 400 sperm per mouse were analyzed.

### Analysis of sperm membrane integrity

#### Eosin-Nigrosin staining

To analyze sperm membrane integrity, 50 μl of sperm swim-out and 50 μl EN stain [0.67 g Eosin Y (color index 45380), 0.9 g sodium chloride, 10 g Nigrosin (color index 50420) and 100 ml ddH_2_O] were mixed and incubated for 30 s. Then, 30 μl of the mix was pipetted onto microscope slides, smeared and mounted with Entellan. In total, 200 sperm per animal were analyzed.

#### Hypoosmotic swelling test

For the HOS test, 100 μl of sperm swim-out was mixed with 1 ml pre-warmed HOS solution (1.375 g D-fructose, 0.75 g sodium citrate dihydrate, 100 ml ddH_2_O) and incubated for 30 min at 37°C. The solution was dropped onto a microscope slide, covered with a cover slip and analyzed within 1 h. In total, 200 sperm per animal were evaluated.

### RNA-sequencing and differential expression analysis

RNA was extracted from whole testis of three individuals per genotype. After removal of the tunica albuginea, testes were homogenized in TRIzol (Thermo Fisher Scientific) and processed according to the manufacturer’s protocol. RNA integrity (RIN) was determined using the RNA Nano 6000 Assay Kit with the Agilent Bioanalyzer 2100 system (Agilent Technologies). RIN values were >7 for all samples. RNA sample quality control, library preparation (QuantSeq 3′-mRNA Library Prep; Lexogen) and RNA-sequencing were performed by the University of Bonn Core Facility for Next Generation Sequencing (NGS). Sequencing was performed on the Illumina HiSeq 2500 V4 platform, producing >10 million, 50 bp 3′-end reads per sample.

Samples were mapped to the mouse genome (GRCm38.89) using HISAT2 2.1 (Kim et al., 2015) and transcripts were quantified and annotated using StringTie 1.3.3 (Pertea et al., 2015). Gene annotation was retrieved from the Ensembl FTP server (ftp://ftp.ensembl.org) (GRCm38.89). The Python script (preDE.py) included in the StringTie package was used to prepare DEseq2-compatible gene-level count matrices for analysis of differential gene expression. Mapping to the *Prm1* genomic location was visualized using the Integrative Genomics Viewer (IGV) (Robinson et al., 2011).

Differential expression (DE) was analyzed using DESeq2 1.16.1 (Love et al., 2014). The adjusted *P*-value (Benjamini–Hochberg method) cut-off for DE was set at <0.05; the log2-fold change (LFC) in expression cut-off was set at >1. We performed Gene Ontology (GO) term and pathway over-representation analyses on relevant lists of genes using the PANTHER gene list analysis tool with Fisher's exact test and false discovery rate (FDR) correction (Mi et al., 2017).

### Mass spectrometry and differential protein abundance analysis

Sperm basic nuclear proteins from three WT, three *Prm1*^−/−^ and three *Prm2*^−/−^ mice were isolated as described below and used for mass spectrometric analysis. Peptide preparation, liquid chromatography (LC)-mass spectrometry (MS) and differential abundance (DA) analysis were performed at the University of Bonn Core Facility Mass Spectrometry.

For peptide preparation, protein solutions (5.5 M urea, 20% 2-mercaptoethanol, 5% acetic acid) were dried in a vacuum concentrator and subjected to in-solution preparation of peptides as described previously (Arévalo et al., 2022). Briefly, cysteines were alkylated with acrylamide and digested with trypsin, followed by desalting.

LC-MS measurements were performed according to Arévalo et al. (2022). Briefly, peptides were separated on a self-packed reversed-phase column within a 90 min gradient. Peptide ions were analyzed with an Orbitrap Lumos mass spectrometer in data-dependent mode with a top-speed method. Precursors and fragment ions were recorded with the Orbitrap detector.

Raw data processing was performed with Proteome Discoverer software in combination with Mascot server version 2.6.1 using *Mus musculus* sequences from SwissProt (2021/03, including isoforms), and contaminants [cRAP (Mellacheruvu et al., 2013)]. Mascot results were filtered for 1% FDR on the basis of q-values from the percolator algorithm (Käll et al., 2008). Spectra with identifications <1% q-value were sent for a second round of database search with semi-tryptic enzyme specificity. Summed abundances were used for relative quantification.

Differential abundance (DA) analysis was performed using the Bioconductor package proDA (Ahlmann-Eltze and Anders, 2021 preprint) using peptide spectrum match (PSM)-level data extracted from Protein Discoverer. Only proteins detected in all genotypes and all replicates with more than two peptides were included in the analysis. The data were log2 transformed and median normalized prior to DA analysis to ensure comparability. The proDA package is based on linear models and uses Bayesian priors to increase power for DA detection (Ahlmann-Eltze and Anders, 2021 preprint). Proteins with a LFC >1 and FDR adjusted *P*<0.05 were considered differentially abundant compared with WT. Plots were generated using the R-package ggplot2 (Wickham, 2011).

### Sperm nuclear morphology analysis

Epididymal sperm were analyzed using the ImageJ plugin ‘Nuclear_Morphology_Analysis_1.18.1_standalone’ (Skinner et al., 2019) as described previously (Schneider et al., 2020). In brief, sperm were fixed in Carnoy's solution, spread on slides, mounted with ROTI Mount FluorCare DAPI and imaged at 100× magnification. A minimum of 100 sperm heads per sample from four biological replicates were analyzed. Testicular sperm on slides were imaged at 63× magnification and a minimum of 120 sperm per genotype were analyzed.

### Isolation of testicular sperm

Testicular sperm were prepared and isolated as described previously (Kotaja et al., 2004). In brief, testes were decapsulated and immersed in PBS. The tubules were separated and pulled apart. Using a Leica MS5 stereomicroscope and a Schott KL1500 light source, tubule segments with the highest light absorption were selected and cut into pieces. Selected pieces were transferred to a glass slide using a pipette and covered with a cover slip. Slides were frozen in liquid nitrogen and the cover slip was flipped off. Cells were fixed for 5 min in Carnoy’s solution, stained with ROTI Mount FluorCare DAPI and covered with a cover slip.

### Sperm motility analysis

Epididymal sperm swim-out was performed in 1 ml sterile filtered THY medium (138 mM NaCl, 4.8 mM KCl, 2 mM CaCl_2_, 1.2 mM KH_2_PO_4_, 1 mM MgSO_4_, 5.6 mM glucose, 10 mM HEPES, 0.5 mM sodium pyruvate, 10 mM L-lactate, pH 7.4, 310-320 mOsm) for 15 min at 37°C. Next, sperm were diluted 1:20-1:50 in dilution medium (3 mg/ml bovine serum albumin in THY medium). Then, 30 µl of the dilution were pipetted onto a glass slide equipped with a spacer and cover slip, placed on a heated slide holder (37°C) and analyzed under an inverted microscope (Leica, DM-IRB) equipped with a camera (acA1920-155ucMED; Basler). The movement of sperm was recorded at 100 frames/s for 3 s and analyzed in ImageJ. The produced ‘z project’ was used to distinguish and count moving and non-moving sperm (*n*=100 sperm/mouse).

### Analysis of sperm basic proteins

Isolation of sperm nuclear proteins was performed according to Soler-Ventura et al. (2018). Briefly, sperm were counted, washed in PBS, pelleted and resuspended in 200 µl buffer containing 4 µl 1 M Tris pH 8, 0.8 µl 0.5 M MgCl_2_ and 5 µl Triton X-100. After centrifugation at 8940 ***g*** for 5 min at 4°C, the pellet was mixed with 1 mM phenylmethylsulfonyl fluoride (PMSF). Lysed cells were mixed with solution 1 containing PMSF and EDTA (10 mM PMSF, 2 mM EDTA, 100 mM Tris, pH 8) and solution 2 containing DTT and GuHCl (0.04435 g in 0.5 ml 6 M GuHCl) and vinylpyridine (0.8%) and incubated for 30 min at 37°C. The addition of 5× ice-cold 100% ethanol precipitated any DNA. Proteins were dissolved in 0.5 M HCl and precipitated with 100% trichloroacetic acid at 4°C. After two acetone washes, the proteins were lyophilized and resuspended in sample buffer (5.5 M urea, 20% 2-mercaptoethanol, 5% acetic acid).

Next, the basic proteins were separated on a pre-electrophorized 15% AU-PAGE (2.5 M urea, 0.9 M acetic acid, 15% acrylamide/ 0.1% *N*,*N*′-methylene *bis*-acrylamide, tetramethylethylenediamine and ammonium persulfate) and visualized with Coomassie Brilliant Blue. Quantification was performed using ImageJ as described previously (Arévalo et al., 2022).

The recombinant (human) histones H1, H2A, H2B, TH2B, H3.1 and H3.3 (Active Motif; 81126, 31490, 31492, 31577, 31294 and 31295, respectively) (1 µg per well) were loaded on a 15% AU-PAGE gel together with WT sperm basic extracts and visualized with Coomassie Brilliant Blue.

### Immunoblotting

Basic protein extracts (equivalent to 1.5 million sperm per well) were separated on 15% AU-PAGE and transferred to PVDF membranes using the Trans-Blot Turbo System (Bio-Rad). Membranes were blocked in 1:1 TBS-0.1% Tween 20 (TBST) ChemiBLOCKER (Sigma-Aldrich/Merck) for 1 h at room temperature. Primary antibodies [ODF2 (Proteintech; 12058-1-AP; 1:500), GPX4 (Abcam; ab125066; 1:1000), H3 (Abcam; ab1791; 1:1000), H2A.L.2 (Govin et al., 2007; 1:1000), SPAG8 (Proteintech; 13915-1-AP; 1:500), PRM1 (1:1000), PRM2 (1:1000)] were diluted in blocking solution and membranes were incubated at 4°C overnight. After washing in TBST, the membranes were incubated with secondary antibodies [polyclonal goat anti-rabbit IgG/HRP (Agilent Technologies/Dako; P044801-2; 1:2000), polyclonal rabbit anti-mouse IgG/HRP (Agilent Technologies/Dako; P026002-2; 1:1000)] for 1 h at room temperature. Following washing in TBST, the signals were detected using WESTAR NOVA 2.0 chemiluminescent substrate (Cyanagen) and the ChemiDoc MP Imaging system (Bio-Rad).

### Statistical analysis

Values are presented as means±standard deviation (s.d.) unless otherwise indicated. Statistical significance was calculated using a two-tailed, unpaired Student's *t*-test and a value of *P*<0.05 was considered significant (*P<0.05; **P<0.005; ***P<0.001).

## Supplementary Material

Supplementary information

Reviewer comments

## References

[DEV200330C1] Ahlmann-Eltze, C. and Anders, S. (2021). proDA: probabilistic dropout analysis for identifying differentially abundant proteins in label-free mass spectrometry. *bioRxiv*. 10.21203/rs.3.rs-36351/v1

[DEV200330C2] Alahmar, A. T. (2019). Role of oxidative stress in male infertility: an updated review. *J. Hum. Reprod. Sci.* 12, 4-18. 10.4103/jhrs.JHRS_150_18 31007461 PMC6472207

[DEV200330C3] Aoki, V. W., Moskovtsev, S. I., Willis, J., Liu, L., Mullen, J. B. and Carrell, D. T. (2005). DNA integrity is compromised in protamine-deficient human sperm. *J. Androl.* 26, 741-748. 10.2164/jandrol.05063 16291969

[DEV200330C4] Arévalo, L., Merges, G. E., Schneider, S., Oben, F. E., Neumann, I. and Schorle, H. (2022). Loss of the cleaved-protamine 2 domain leads to incomplete histone-to-protamine exchange and infertility in mice. *PLoS Genet.* (in press). 10.1101/2021.09.29.462440 PMC9273070 35763544

[DEV200330C5] Balhorn, R. (1982). A model for the structure of chromatin in mammalian sperm. *J. Cell Biol.* 93, 298-305. 10.1083/jcb.93.2.298 7096440 PMC2112839

[DEV200330C6] Balhorn, R. (2007). The protamine family of sperm nuclear proteins. *Genome Biol.* 8, 227. 10.1186/gb-2007-8-9-227 17903313 PMC2375014

[DEV200330C7] Balhorn, R., Reed, S. and Tanphaichitr, N. (1988). Aberrant protamine 1/protamine 2 ratios in sperm of infertile human males. *Experientia* 44, 52-55. 10.1007/BF01960243 3350120

[DEV200330C8] Barral, S., Morozumi, Y., Tanaka, H., Montellier, E., Govin, J., De Dieuleveult, M., Charbonnier, G., Coute, Y., Puthier, D., Buchou, T. et al. (2017). Histone variant H2A.L.2 guides transition protein-dependent protamine assembly in male germ cells. *Mol. Cell* 66, 89-101.e8. 10.1016/j.molcel.2017.02.025 28366643

[DEV200330C9] Belokopytova, I. A., Kostyleva, E. I., Tomilin, A. N. and Vorob'ev, V. I. (1993). Human male infertility may be due to a decrease of the protamine P2 content in sperm chromatin. *Mol. Reprod. Dev.* 34, 53-57. 10.1002/mrd.1080340109 8418817

[DEV200330C10] Bench, G., Corzett, M. H., De Yebra, L., Oliva, R. and Balhorn, R. (1998). Protein and DNA contents in sperm from an infertile human male possessing protamine defects that vary over time. *Mol. Reprod. Dev.* 50, 345-353. 10.1002/(SICI)1098-2795(199807)50:3<345::AID-MRD11>3.0.CO;2-3 9621311

[DEV200330C11] Chauviere, M., Martinage, A., Debarle, M., Sautiere, P. and Chevaillier, P. (1992). Molecular characterization of six intermediate proteins in the processing of mouse protamine P2 precursor. *Eur. J. Biochem.* 204, 759-765. 10.1111/j.1432-1033.1992.tb16691.x 1541289

[DEV200330C12] Cho, C., Willis, W. D., Goulding, E. H., Jung-Ha, H., Choi, Y.-C., Hecht, N. B. and Eddy, E. M. (2001). Haploinsufficiency of protamine-1 or -2 causes infertility in mice. *Nat. Genet.* 28, 82-86. 10.1038/ng0501-82 11326282

[DEV200330C13] Cho, C., Jung-Ha, H., Willis, W. D., Goulding, E. H., Stein, P., Xu, Z., Schultz, R. M., Hecht, N. B. and Eddy, E. M. (2003). Protamine 2 deficiency leads to sperm DNA damage and embryo death in mice. *Biol. Reprod.* 69, 211-217. 10.1095/biolreprod.102.015115 12620939

[DEV200330C14] Cooper, T. G., Noonan, E., Von Eckardstein, S., Auger, J., Baker, H. W. G., Behre, H. M., Haugen, T. B., Kruger, T., Wang, C., Mbizvo, M. T. et al. (2010). World Health Organization reference values for human semen characteristics. *Hum. Reprod. Update* 16, 231-245. 10.1093/humupd/dmp048 19934213

[DEV200330C15] Corzett, M., Mazrimas, J. and Balhorn, R. (2002). Protamine 1: protamine 2 stoichiometry in the sperm of eutherian mammals. *Mol. Reprod. Dev.* 61, 519-527. 10.1002/mrd.10105 11891924

[DEV200330C16] De Lamirande, E., Jiang, H., Zini, A., Kodama, H. and Gagnon, C. (1997). Reactive oxygen species and sperm physiology. *Rev. Reprod.* 2, 48-54. 10.1530/ror.0.0020048 9414465

[DEV200330C17] De Mateo, S., Gázquez, C., Guimerà, M., Balasch, J., Meistrich, M. L., Ballescà, J. L. and Oliva, R. (2009). Protamine 2 precursors (Pre-P2), protamine 1 to protamine 2 ratio (P1/P2), and assisted reproduction outcome. *Fertil. Steril.* 91, 715-722. 10.1016/j.fertnstert.2007.12.047 18314125

[DEV200330C18] De Yebra, L., Ballesca, J. L., Vanrell, J. A., Corzett, M., Balhorn, R. and Oliva, R. (1998). Detection of P2 precursors in the sperm cells of infertile patients who have reduced protamine P2 levels. *Fertil. Steril.* 69, 755-759. 10.1016/S0015-0282(98)00012-0 9548169

[DEV200330C19] García-Peiró, A., Martínez-Heredia, J., Oliver-Bonet, M., Abad, C., Amengual, M. J., Navarro, J., Jones, C., Coward, K., Gosálvez, J. and Benet, J. (2011). Protamine 1 to protamine 2 ratio correlates with dynamic aspects of DNA fragmentation in human sperm. *Fertil. Steril.* 95, 105-109. 10.1016/j.fertnstert.2010.06.053 20667534

[DEV200330C20] Gawlik, V., Schmidt, S., Scheepers, A., Wennemuth, G., Augustin, R., Aumüller, G., Moser, M., Al-Hasani, H., Kluge, R., Joost, H. G. et al. (2008). Targeted disruption of Slc2a8 (GLUT8) reduces motility and mitochondrial potential of spermatozoa. *Mol. Membr. Biol.* 25, 224-235. 10.1080/09687680701855405 18428038 PMC2557070

[DEV200330C21] Goodson, S. G., Zhang, Z., Tsuruta, J. K., Wang, W. and O'Brien, D. A. (2011). Classification of mouse sperm motility patterns using an automated multiclass support vector machines model. *Biol. Reprod.* 84, 1207-1215. 10.1095/biolreprod.110.088989 21349820 PMC3099585

[DEV200330C22] Govin, J., Caron, C., Escoffier, E., Ferro, M., Kuhn, L., Rousseaux, S., Eddy, E. M., Garin, J. and Khochbin, S. (2006). Post-meiotic shifts in HSPA2/HSP70.2 chaperone activity during mouse spermatogenesis. *J. Biol. Chem.* 281, 37888-37892. 10.1074/jbc.M608147200 17035236 PMC1896149

[DEV200330C66] Govin, J., Escoffier, E., Rousseaux, S., Kuhn, L., Ferro, M., Thevenon, J., Catena, R., Davidson, I., Garin, J., Khochbin, S. et al. (2007). Pericentric heterochromatin reprogramming by new histone variants during mouse spermiogenesis. *J. Cell Biol.* 176, 283-94. 10.1083/jcb.200604141 17261847 PMC2063955

[DEV200330C24] Henricks, D. M. and Mayer, D. T. (1965). Isolation and characterization of a basic keratin-like protein from mammalian spermatozoa. *Exp. Cell Res.* 40, 402-412. 10.1016/0014-4827(65)90273-9 5892636

[DEV200330C25] Käll, L., Storey, J. D., Maccoss, M. J. and Noble, W. S. (2008). Assigning significance to peptides identified by tandem mass spectrometry using decoy databases. *J. Proteome Res.* 7, 29-34. 10.1021/pr700600n 18067246

[DEV200330C26] Khara, K. K., Vlad, M., Griffiths, M. and Kennedy, C. R. (1997). Human protamines and male infertility. *J. Assist. Reprod. Genet.* 14, 282-290. 10.1007/BF02765830 9147242 PMC3454727

[DEV200330C27] Kim, D., Langmead, B. and Salzberg, S. L. (2015). HISAT: a fast spliced aligner with low memory requirements. *Nat. Methods* 12, 357-360. 10.1038/nmeth.3317 25751142 PMC4655817

[DEV200330C28] Koppers, A. J., De Iuliis, G. N., Finnie, J. M., Mclaughlin, E. A. and Aitken, R. J. (2008). Significance of mitochondrial reactive oxygen species in the generation of oxidative stress in spermatozoa. *J. Clin. Endocrinol. Metab.* 93, 3199-3207. 10.1210/jc.2007-2616 18492763

[DEV200330C29] Kotaja, N., Kimmins, S., Brancorsini, S., Hentsch, D., Vonesch, J.-L., Davidson, I., Parvinen, M. and Sassone-Corsi, P. (2004). Preparation, isolation and characterization of stage-specific spermatogenic cells for cellular and molecular analysis. *Nat. Methods* 1, 249-254. 10.1038/nmeth1204-249 16144087

[DEV200330C30] Krawetz, S. A. and Dixon, G. H. (1988). Sequence similarities of the protamine genes: implications for regulation and evolution. *J. Mol. Evol.* 27, 291-297. 10.1007/BF02101190 3146639

[DEV200330C31] Lolis, D., Georgiou, I., Syrrou, M., Zikopoulos, K., Konstantelli, M. and Messinis, I. (1996). Chromomycin A3-staining as an indicator of protamine deficiency and fertilization. *Int. J. Androl.* 19, 23-27. 10.1111/j.1365-2605.1996.tb00429.x 8698534

[DEV200330C32] Love, M. I., Huber, W. and Anders, S. (2014). Moderated estimation of fold change and dispersion for RNA-seq data with DESeq2. *Genome Biol.* 15, 550. 10.1186/s13059-014-0550-8 25516281 PMC4302049

[DEV200330C33] Lüke, L., Tourmente, M., Dopazo, H., Serra, F. and Roldan, E. R. S. (2016a). Selective constraints on protamine 2 in primates and rodents. *BMC Evol. Biol.* 16, 21. 10.1186/s12862-016-0588-1 26801756 PMC4724148

[DEV200330C34] Lüke, L., Tourmente, M. and Roldan, E. R. S. (2016b). Sexual selection of protamine 1 in mammals. *Mol. Biol. Evol.* 33, 174-184. 10.1093/molbev/msv209 26429923

[DEV200330C35] Mashiko, D., Fujihara, Y., Satouh, Y., Miyata, H., Isotani, A. and Ikawa, M. (2013). Generation of mutant mice by pronuclear injection of circular plasmid expressing Cas9 and single guided RNA. *Sci. Rep.* 3, 3355. 10.1038/srep03355 24284873 PMC3842082

[DEV200330C36] Mellacheruvu, D., Wright, Z., Couzens, A. L., Lambert, J.-P., St-Denis, N. A., Li, T., Miteva, Y. V., Hauri, S., Sardiu, M. E., Low, T. Y. et al. (2013). The CRAPome: a contaminant repository for affinity purification-mass spectrometry data. *Nat. Methods* 10, 730-736. 10.1038/nmeth.2557 23921808 PMC3773500

[DEV200330C37] Mi, H., Huang, X., Muruganujan, A., Tang, H., Mills, C., Kang, D. and Thomas, P. D. (2017). PANTHER version 11: expanded annotation data from Gene Ontology and Reactome pathways, and data analysis tool enhancements. *Nucleic Acids Res.* 45, D183-D189. 10.1093/nar/gkw1138 27899595 PMC5210595

[DEV200330C38] Moritz, L., Schon, S. B., Rabbani, M., Sheng, Y., Pendlebury, D. F., Agrawal, R., Sultan, C., Jorgensen, K., Zheng, X., Diehl, A. et al. (2021). Single residue substitution in protamine 1 disrupts sperm genome packaging and embryonic development in mice. *bioRxiv*. 10.1101/2021.09.16.460631

[DEV200330C39] Ni, K., Spiess, A.-N., Schuppe, H.-C. and Steger, K. (2016). The impact of sperm protamine deficiency and sperm DNA damage on human male fertility: a systematic review and meta-analysis. *Andrology* 4, 789-799. 10.1111/andr.12216 27231200

[DEV200330C40] Oliva, R. (2006). Protamines and male infertility. *Hum. Reprod. Update* 12, 417-435. 10.1093/humupd/dml009 16581810

[DEV200330C41] Pertea, M., Pertea, G. M., Antonescu, C. M., Chang, T. C., Mendell, J. T. and Salzberg, S. L. (2015). StringTie enables improved reconstruction of a transcriptome from RNA-seq reads. *Nat. Biotechnol.* 33, 290-295. 10.1038/nbt.3122 25690850 PMC4643835

[DEV200330C42] Rathke, C., Baarends, W. M., Awe, S. and Renkawitz-Pohl, R. (2014). Chromatin dynamics during spermiogenesis. *Biochim. Biophys. Acta* 1839, 155-168. 10.1016/j.bbagrm.2013.08.004 24091090

[DEV200330C43] Reeves, R. H., Gearhart, J. D., Hecht, N. B., Yelick, P., Johnson, P. and O'Brien, S. J. (1989). Mapping of PRM 1 to human chromosome 16 and tight linkage of Prm-1 and Prm-2 on mouse chromosome 16. *J. Hered.* 80, 442-446. 10.1093/oxfordjournals.jhered.a110895 2614060

[DEV200330C44] Retief, J. D. and Dixon, G. H. (1993). Evolution of pro-protamine P2 genes in primates. *Eur. J. Biochem.* 214, 609-615. 10.1111/j.1432-1033.1993.tb17960.x 8513810

[DEV200330C45] Robinson, J. T., Thorvaldsdóttir, H., Winckler, W., Guttman, M., Lander, E. S., Getz, G. and Mesirov, J. P. (2011). Integrative genomics viewer. *Nat. Biotechnol.* 29, 24-26. 10.1038/nbt.1754 21221095 PMC3346182

[DEV200330C46] Sadeghi, S., Talebi, A. R., Shahedi, A., Moein, M. R. and Abbasi-Sarcheshmeh, A. (2019). Effects of tamoxifen on DNA integrity in mice. *J. Reprod. Infertil.* 20, 10-15. 30859077 PMC6386794

[DEV200330C47] Sakkas, D., Urner, F., Bianchi, P. G., Bizzaro, D., Wagner, I., Jaquenoud, N., Manicardi, G. and Campana, A. (1996). Sperm chromatin anomalies can influence decondensation after intracytoplasmic sperm injection. *Hum. Reprod.* 11, 837-843. 10.1093/oxfordjournals.humrep.a019263 8671337

[DEV200330C48] Schneider, S., Balbach, M., Jan, F. J., Fietz, D., Nettersheim, D., Jostes, S., Schmidt, R., Kressin, M., Bergmann, M., Wachten, D. et al. (2016). Re-visiting the Protamine-2 locus: deletion, but not haploinsufficiency, renders male mice infertile. *Sci. Rep.* 6, 36764. 10.1038/srep36764 27833122 PMC5105070

[DEV200330C49] Schneider, S., Shakeri, F., Trotschel, C., Arevalo, L., Kruse, A., Buness, A., Poetsch, A., Steger, K. and Schorle, H. (2020). Protamine-2 deficiency initiates a Reactive Oxygen Species (ROS)-mediated destruction cascade during epididymal sperm maturation in mice. *Cells* 9, 1789. 10.3390/cells9081789 PMC7463811 32727081

[DEV200330C50] Shirley, C. R., Hayashi, S., Mounsey, S., Yanagimachi, R. and Meistrich, M. L. (2004). Abnormalities and reduced reproductive potential of sperm from Tnp1- and Tnp2-null double mutant mice. *Biol. Reprod.* 71, 1220-1229. 10.1095/biolreprod.104.029363 15189834

[DEV200330C51] Skinner, B. M., Rathje, C. C., Bacon, J., Johnson, E. E. P., Larson, E. L., Kopania, E. E. K., Good, J. M., Yousafzai, G., Affara, N. A. and Ellis, P. J. I. (2019). A high-throughput method for unbiased quantitation and categorization of nuclear morphology. *Biol. Reprod.* 100, 1250-1260. 10.1093/biolre/ioz013 30753283 PMC6497523

[DEV200330C52] Soler-Ventura, A., Castillo, J., De La Iglesia, A., Jodar, M., Barrachina, F., Ballesca, J. L. and Oliva, R. (2018). Mammalian sperm protamine extraction and analysis: a step-by-step detailed protocol and brief review of protamine alterations. *Protein Pept. Lett.* 25, 424-433. 10.2174/0929866525666180412155205 29651936

[DEV200330C53] Steger, K. (1999). Transcriptional and translational regulation of gene expression in haploid spermatids. *Anat. Embryol. (Berl)* 199, 471-487. 10.1007/s004290050245 10350128

[DEV200330C54] Steger, K., Failing, K., Klonisch, T., Behre, H. M., Manning, M., Weidner, W., Hertle, L., Bergmann, M. and Kliesch, S. (2001). Round spermatids from infertile men exhibit decreased protamine-1 and -2 mRNA. *Hum. Reprod.* 16, 709-716. 10.1093/humrep/16.4.709 11278223

[DEV200330C55] Steger, K., Fink, L., Failing, K., Bohle, R. M., Kliesch, S., Weidner, W. and Bergmann, M. (2003). Decreased protamine-1 transcript levels in testes from infertile men. *Mol. Hum. Reprod.* 9, 331-336. 10.1093/molehr/gag041 12771233

[DEV200330C56] Steger, K., Wilhelm, J., Konrad, L., Stalf, T., Greb, R., Diemer, T., Kliesch, S., Bergmann, M. and Weidner, W. (2008). Both protamine-1 to protamine-2 mRNA ratio and Bcl2 mRNA content in testicular spermatids and ejaculated spermatozoa discriminate between fertile and infertile men. *Hum. Reprod.* 23, 11-16. 10.1093/humrep/dem363 18003625

[DEV200330C57] Takeda, N., Yoshinaga, K., Furushima, K., Takamune, K., Li, Z., Abe, S., Aizawa, S. and Yamamura, K. (2016). Viable offspring obtained from Prm1-deficient sperm in mice. *Sci. Rep.* 6, 27409. 10.1038/srep27409 27250771 PMC4890041

[DEV200330C58] Torregrosa, N., Domínguez-Fandos, D., Camejo, M. I., Shirley, C. R., Meistrich, M. L., Ballesca, J. L. and Oliva, R. (2006). Protamine 2 precursors, protamine 1/protamine 2 ratio, DNA integrity and other sperm parameters in infertile patients. *Hum. Reprod.* 21, 2084-2089. 10.1093/humrep/del114 16632464

[DEV200330C59] Weyrich, A. (2012). Preparation of genomic DNA from mammalian sperm. *Curr. Protoc. Mol. Biol.* 98, Chapter 2, Unit 2.13.1-3. 10.1002/0471142727.mb0213s98 22470062

[DEV200330C60] Wickham, H. (2011). ggplot2. *Wiley Interdisciplinary Reviews: Computational Statistics*, 3, 180-185.

[DEV200330C61] World Health Organization. (2010). *WHO Laboratory Manual for the Examination and Processing of Human Semen*, 5th edn. Geneva: World Health Organization.

[DEV200330C62] Wykes, S. M. and Krawetz, S. A. (2003). Conservation of the PRM1→PRM2→TNP2 domain. *DNA Seq* 14, 359-367. 10.1080/10425170310001599453 14756422

[DEV200330C63] Yelick, P. C., Balhorn, R., Johnson, P. A., Corzett, M., Mazrimas, J. A., Kleene, K. C. and Hecht, N. B. (1987). Mouse protamine 2 is synthesized as a precursor whereas mouse protamine 1 is not. *Mol. Cell. Biol.* 7, 2173-2179. 10.1128/mcb.7.6.2173-2179.1987 3600661 PMC365340

[DEV200330C64] Zandemami, M., Qujeq, D., Akhondi, M. M., Kamali, K., Raygani, M., Lakpour, N., Shiraz, E. S. and Sadeghi, M. R. (2012). Correlation of CMA3 staining with sperm quality and protamine deficiency. *Lab. Med.* 43, 262-267. 10.1309/LMB42F9QXYKFLJNG

[DEV200330C65] Zhao, M., Shirley, C. R., Yu, Y. E., Mohapatra, B., Zhang, Y., Unni, E., Deng, J. M., Arango, N. A., Terry, N. H. A., Weil, M. M. et al. (2001). Targeted disruption of the transition protein 2 gene affects sperm chromatin structure and reduces fertility in mice. *Mol. Cell. Biol.* 21, 7243-7255. 10.1128/MCB.21.21.7243-7255.2001 11585907 PMC99899

