# Supplementary material for: Loss of Prm1 leads to defective chromatin protamination, impaired PRM2 processing, reduced sperm motility and subfertility in male mice
Source: Development. 2022 Jun 17;149(12):dev200330. doi: 10.1242/dev.200330 (PMC9270976; doi:10.1242/dev.200330)
Supplement: Supplementary information [file develop-149-200330-s1.pdf]

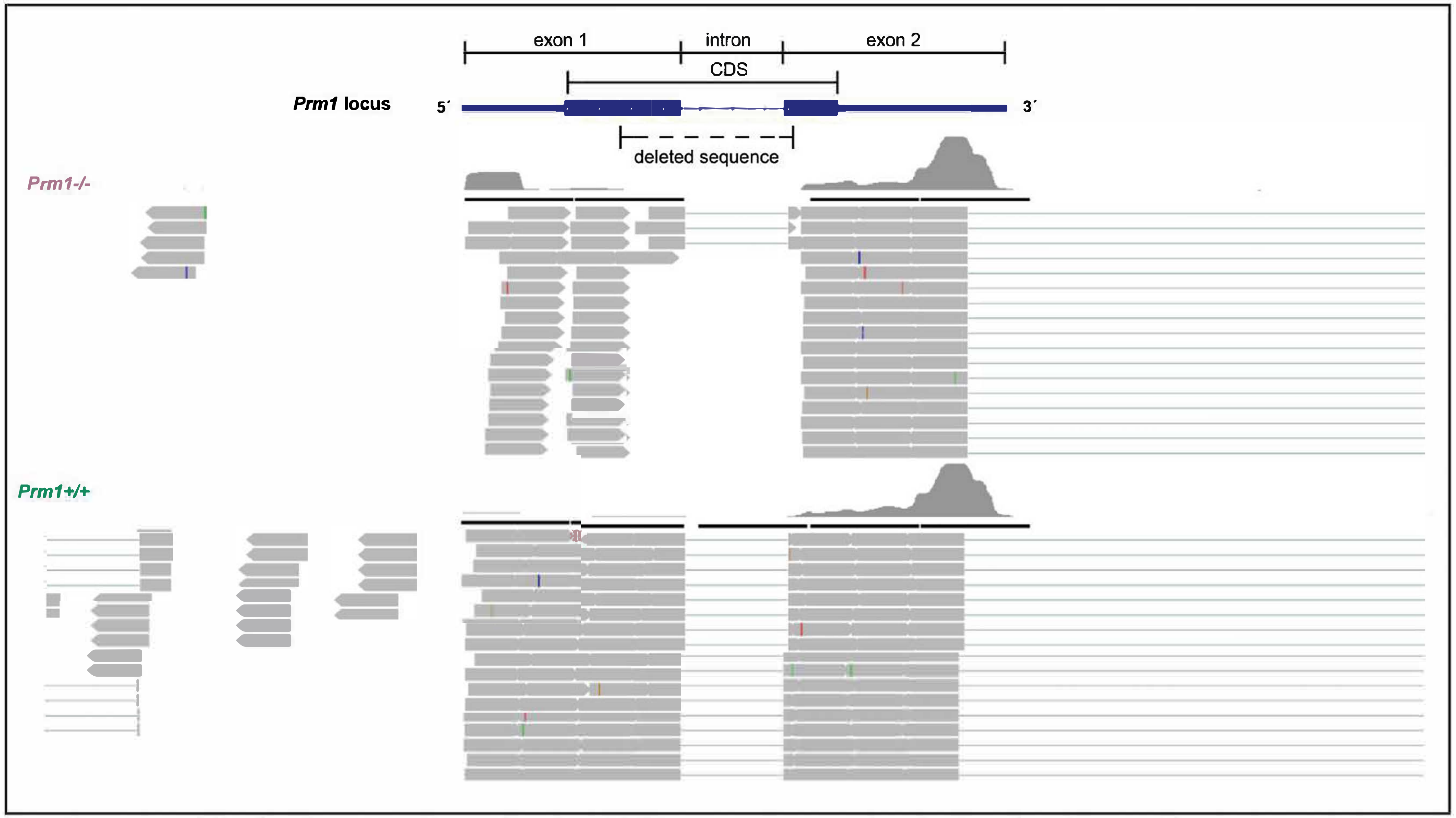

Fig. S1. Represenative cut-out of RNAseq reads mapping to the *Prm1* locus. Reads **from** whole testis RNAseq **of** *Prm1*+/+ and *Prm1*-/- mice.

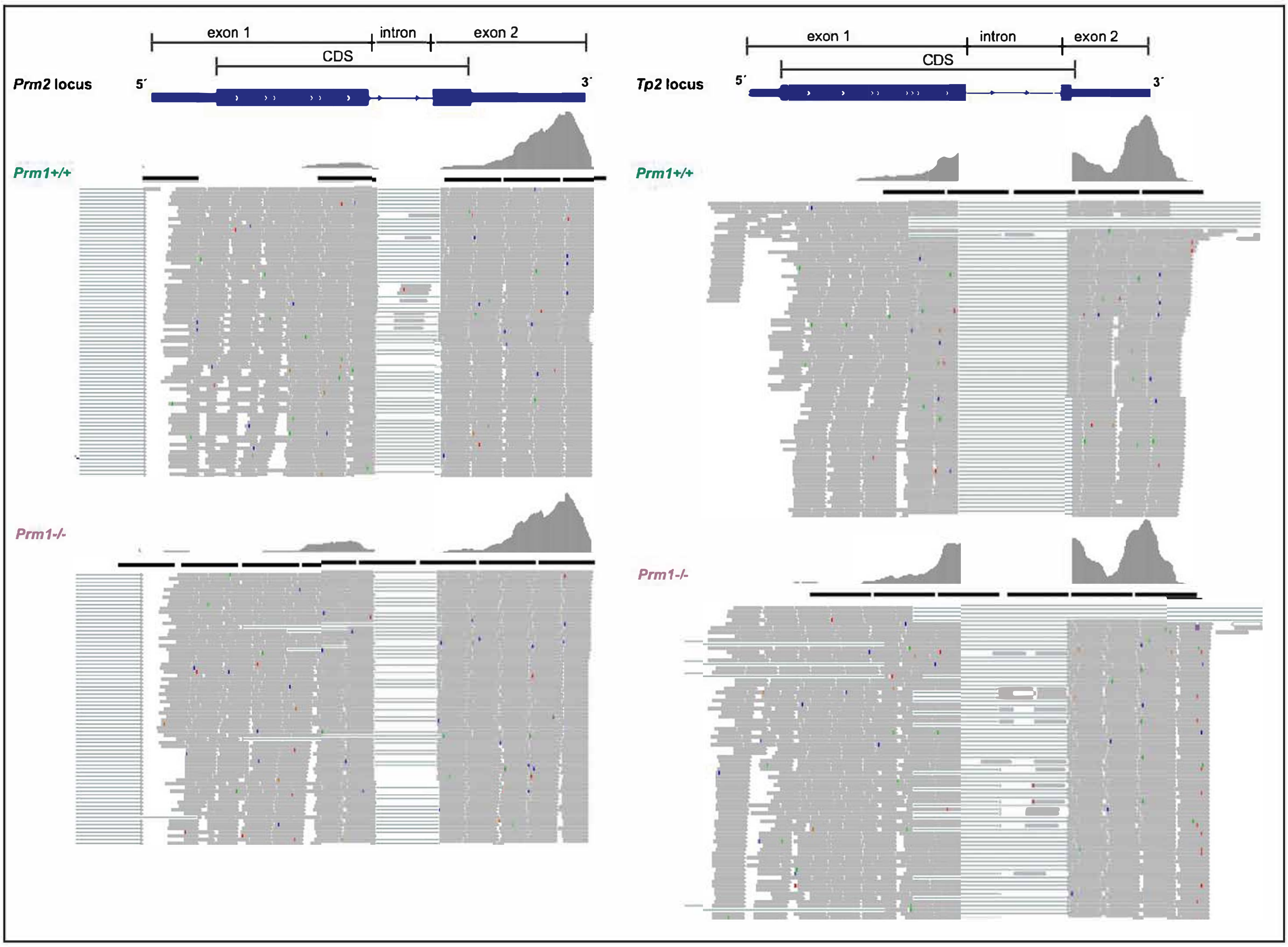

Fig.S2.. Represenative cut-out of RNAseq reads mapping to the *Prm2* and *Tp2* locus. Reads from whole testis RNAseq of *Prm1+/+* and *Prm1-/-* mice.

Development • Supplementary information

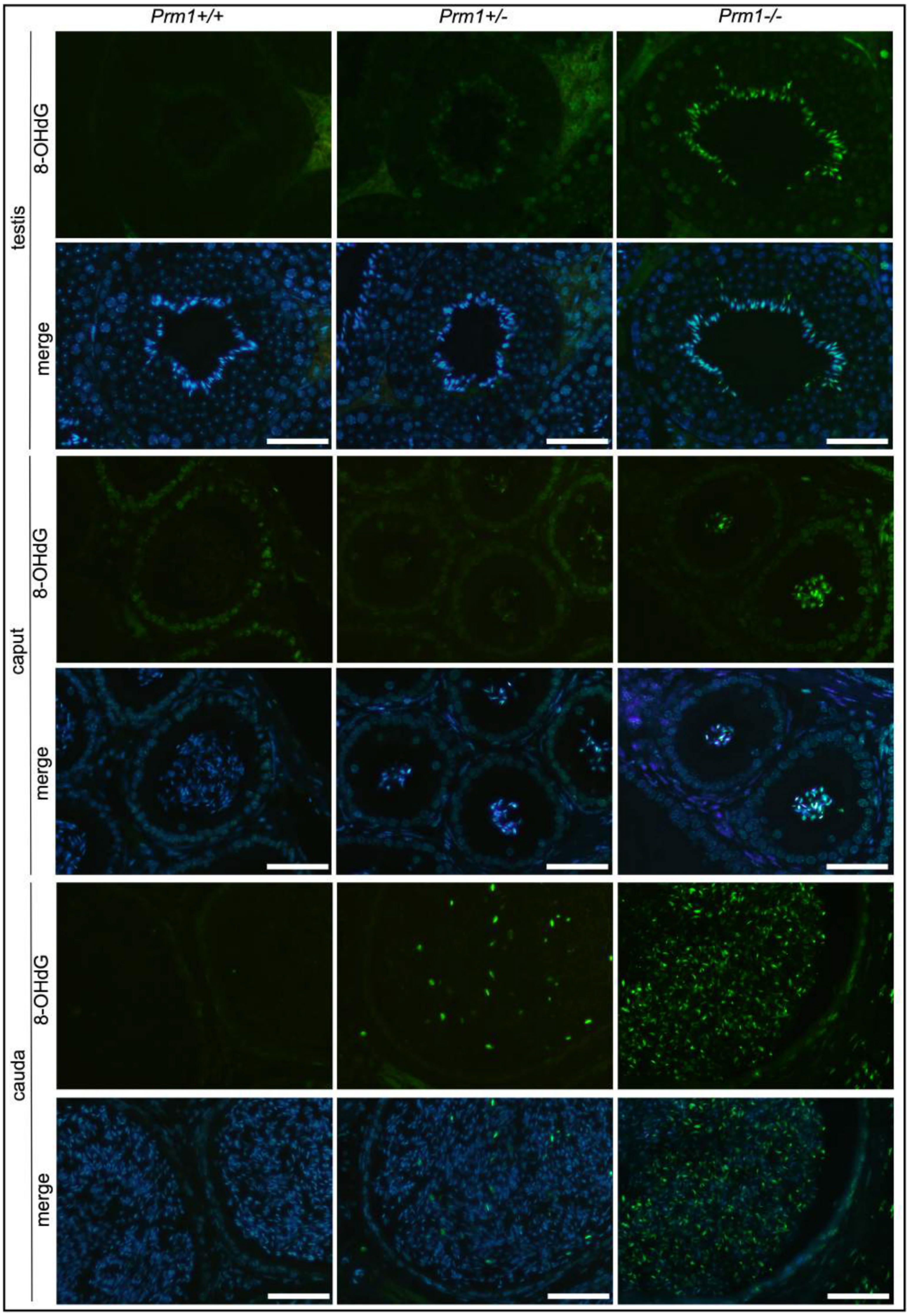

Fig. S3. Representative immunofluorescent staining against 8-OHdG. Testis, caput epididymis and cauda epididymis tissue sections from *Prm1+/+*, *Prm1+/-* and *Prm1-/-* males are shown. Single green and merged channels are shown. Scale: 50 µm

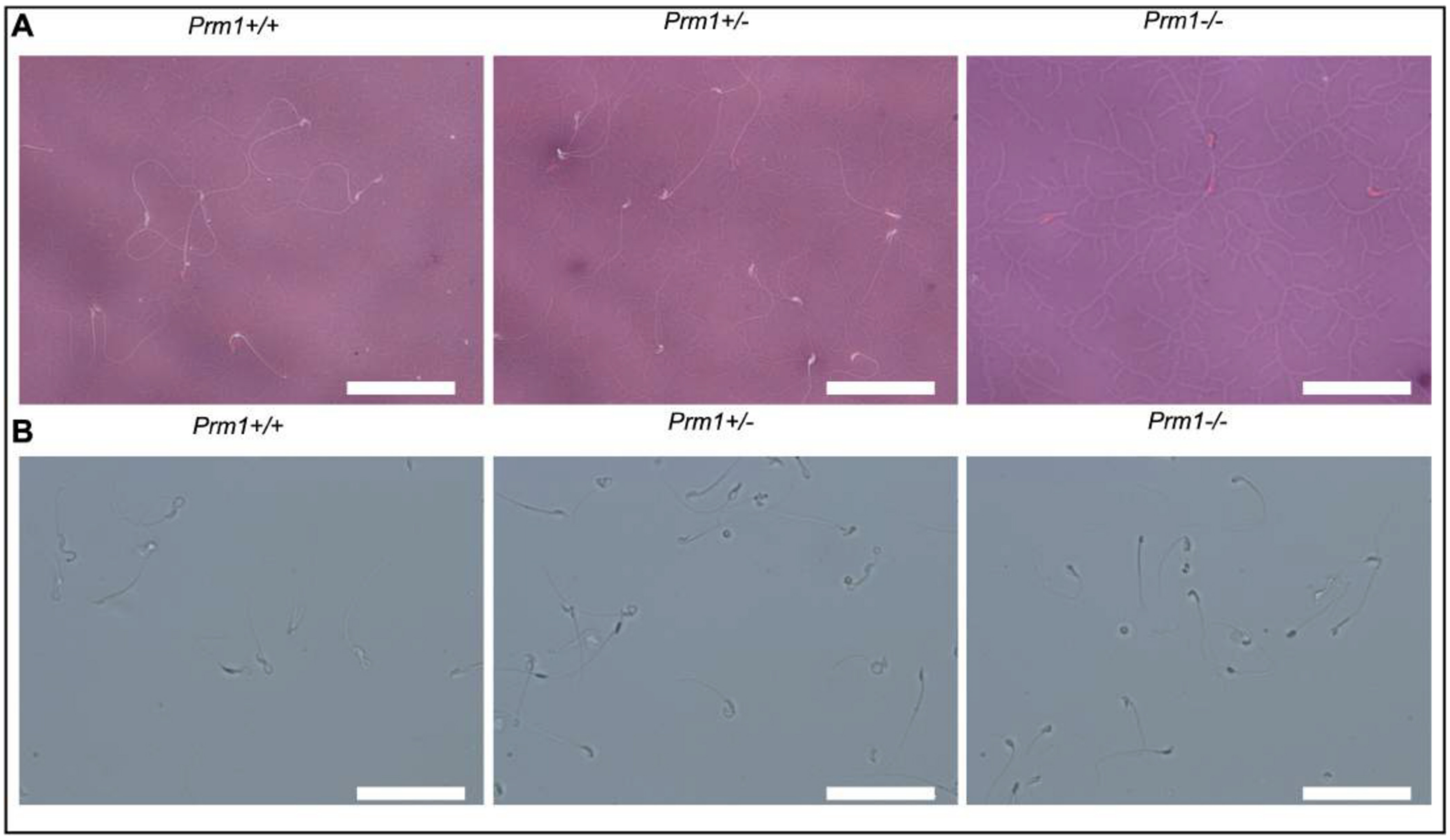

Fig. S4. Membrane damages of Prm1-deficient sperm. (a) Representative pictures of Eosin-Nigrosin staining of *Prm1+/+, Prm1+/-* and *Prm1-/-* sperm. Scale: 50 µm **(b)** Representative pictures of hypoosmotic swelling tests of *Prm1+/+, Prm1+/-* and *Prm1-/-* sperm. Scale: 50 µm

Development • Supplementary information

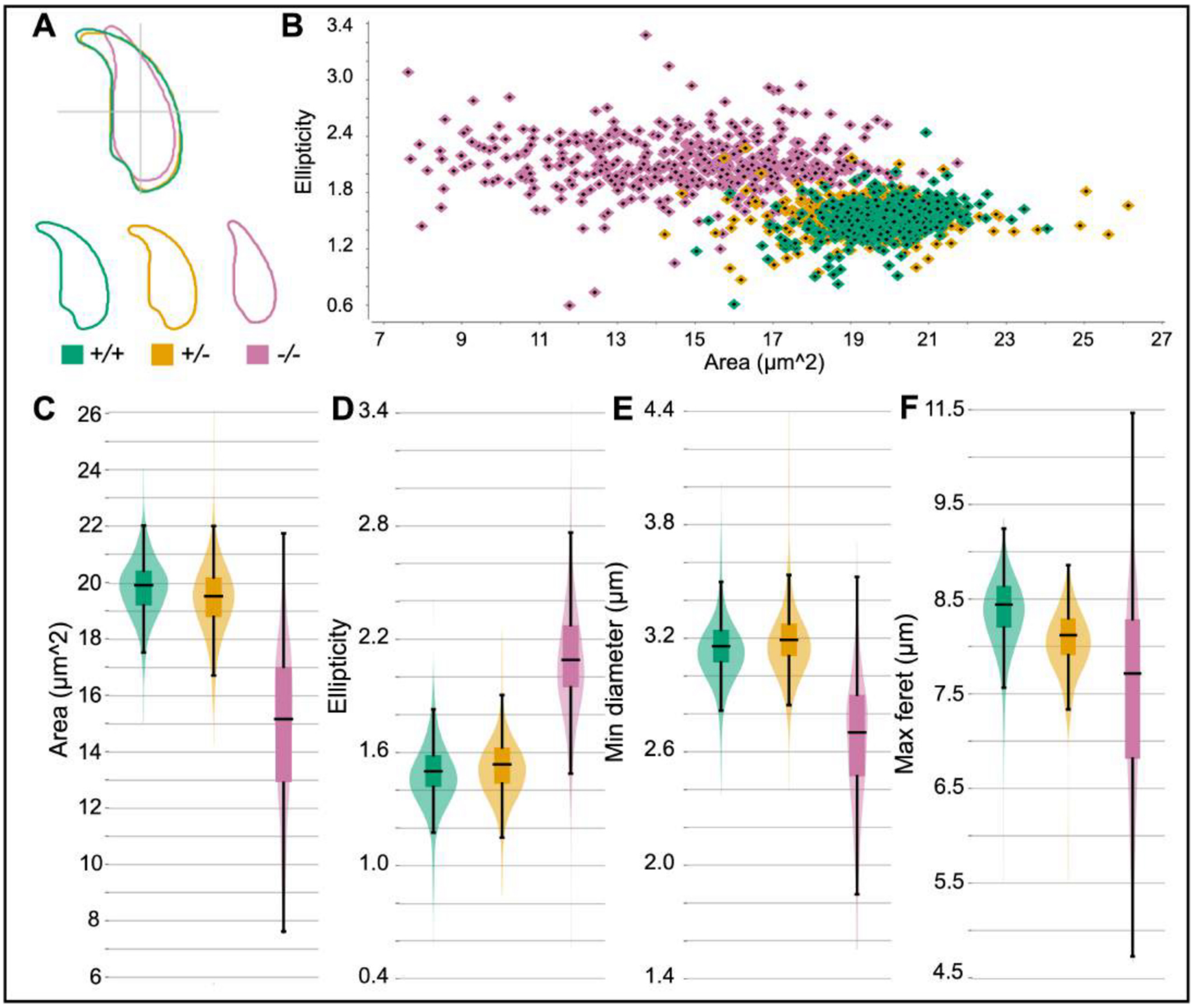

Fig. S5. Nuclear head morphology analysis of epididymal *Prm1*-deficient sperm. (A) Consensus head shapes for epididymal sperm from *Prm1* +/+, *Prm1+/-* and *Prm1-/-* animals are depicted. **(B)** Scatter plot depicting the sperm head shapes of *Prm1+/+, Prm1+/-* and *Prm1-I-* mice by area (µm"2) and ellipticity (bonding heighU bonding width). **(C)** Violin plot presenting the mean area (µm"2) of *Prm1+/+, Prm1+/-* and *Prm1-/-* sperm heads. **(D)** Violin plot showing the mean ellipticity (bonding heighU bonding width) of *Prm1+/+, Prm1+/-* and *Prm1-I-* sperm heads. **(E)** Violin plot showing the mean minimum diameter (µm) of *Prm1+/+, Prm1+/-* and *Prm1-/-* sperm heads. **(F)** Violin plot depicting the mean maximum ferret (µm) of *Prm1+/+, Prm1+/-* and *Prm1-I-* sperm heads.

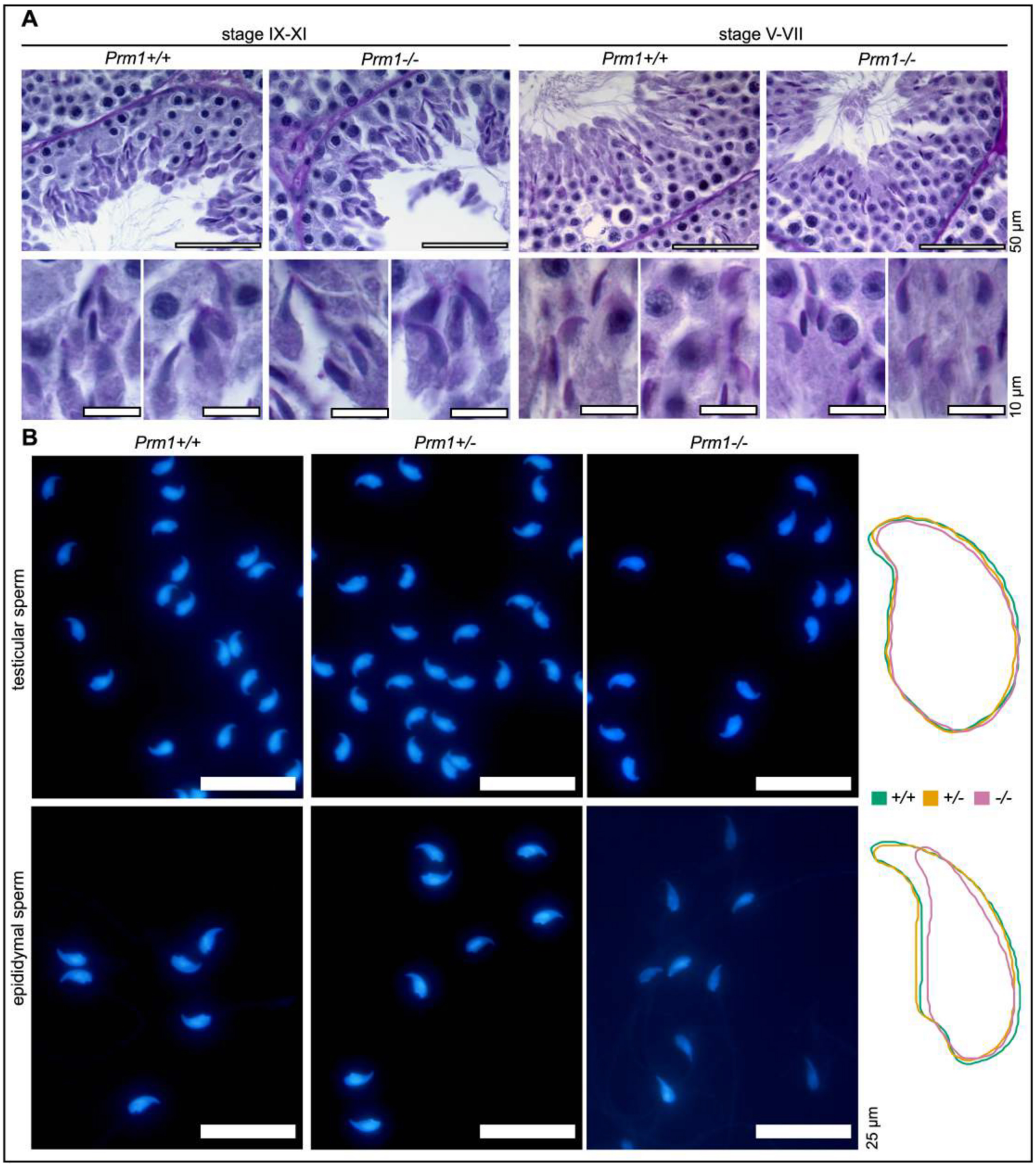

Fig. S6. Head shape analysis of *Prm1+/+* and *Prm1-/-* sperm. (A) Representative Periodic acid-Schiff staings of *Prm1+/+* and *Prm1-/-* seminiferous tubules. Scale: 50 µm and 10 µm. **(B)** DAPI-stained fixed testicular and cauda epididymal sperm from *Prm1+/+, Prm1+/-* and *Prm1-/-* mice. Scale: 25 µm. The consensus shape for each population analysed is depicted.

Development • Supplementary information

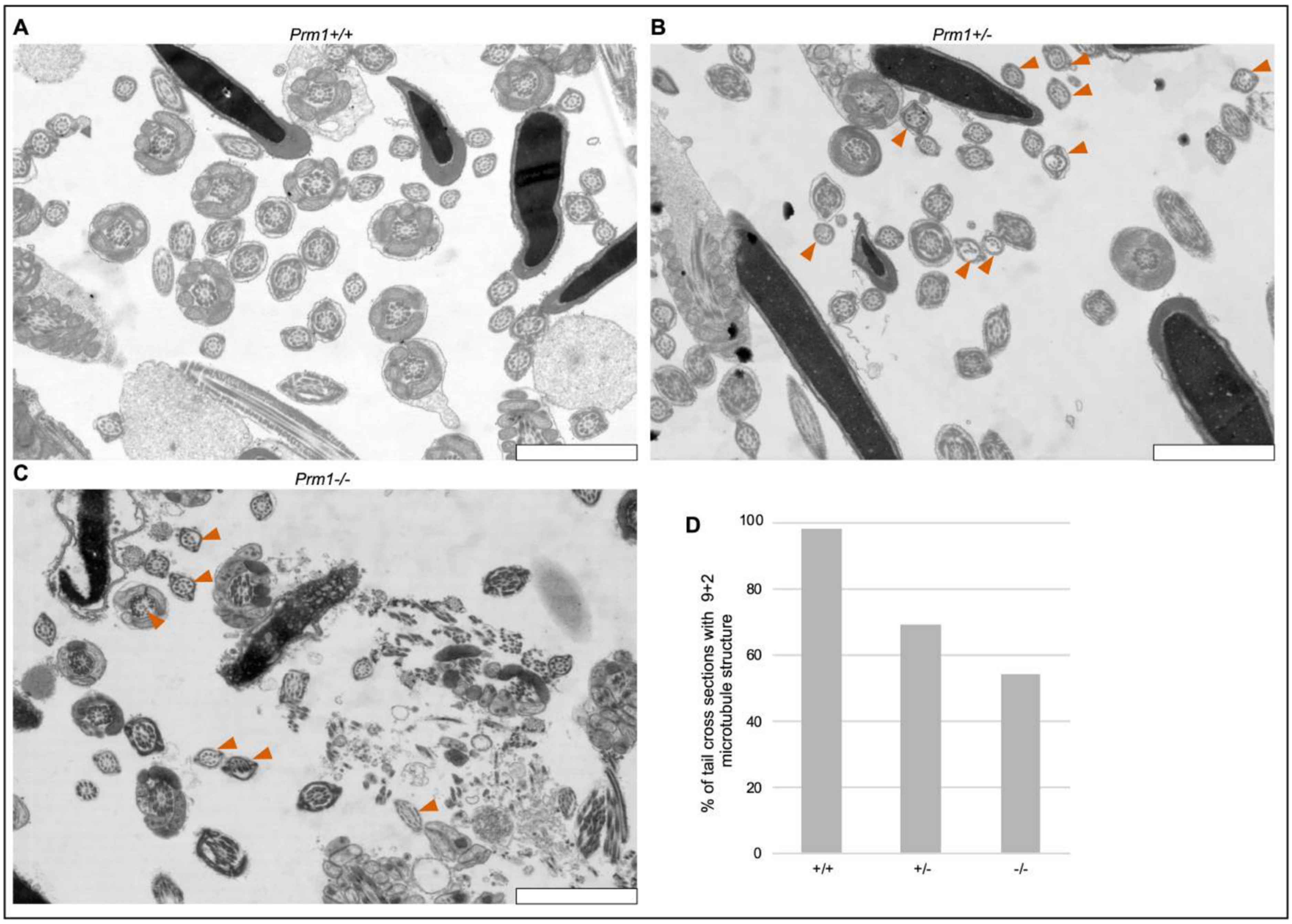

Fig. S7. Sperm flagellar structure of *Prm1-deficient* mice. (A-C) Represenstative transmission electron micrographs of *Prm1+/+*, *Prm1+/-* and *Prm1-l-* epididymal sperm, respectively. Abnormal are indicated by vermillion arrow heads. Scale: 2 µm. **(D)** Quantification of sperm flagella crosssections with correct 9+2 microtuble structure of *Prm1+/+*, *Prm1+/-* and *Prm1-/-* mice. A minimum of 400 tail cross sections per genotype were counted.

Development • Supplementary information

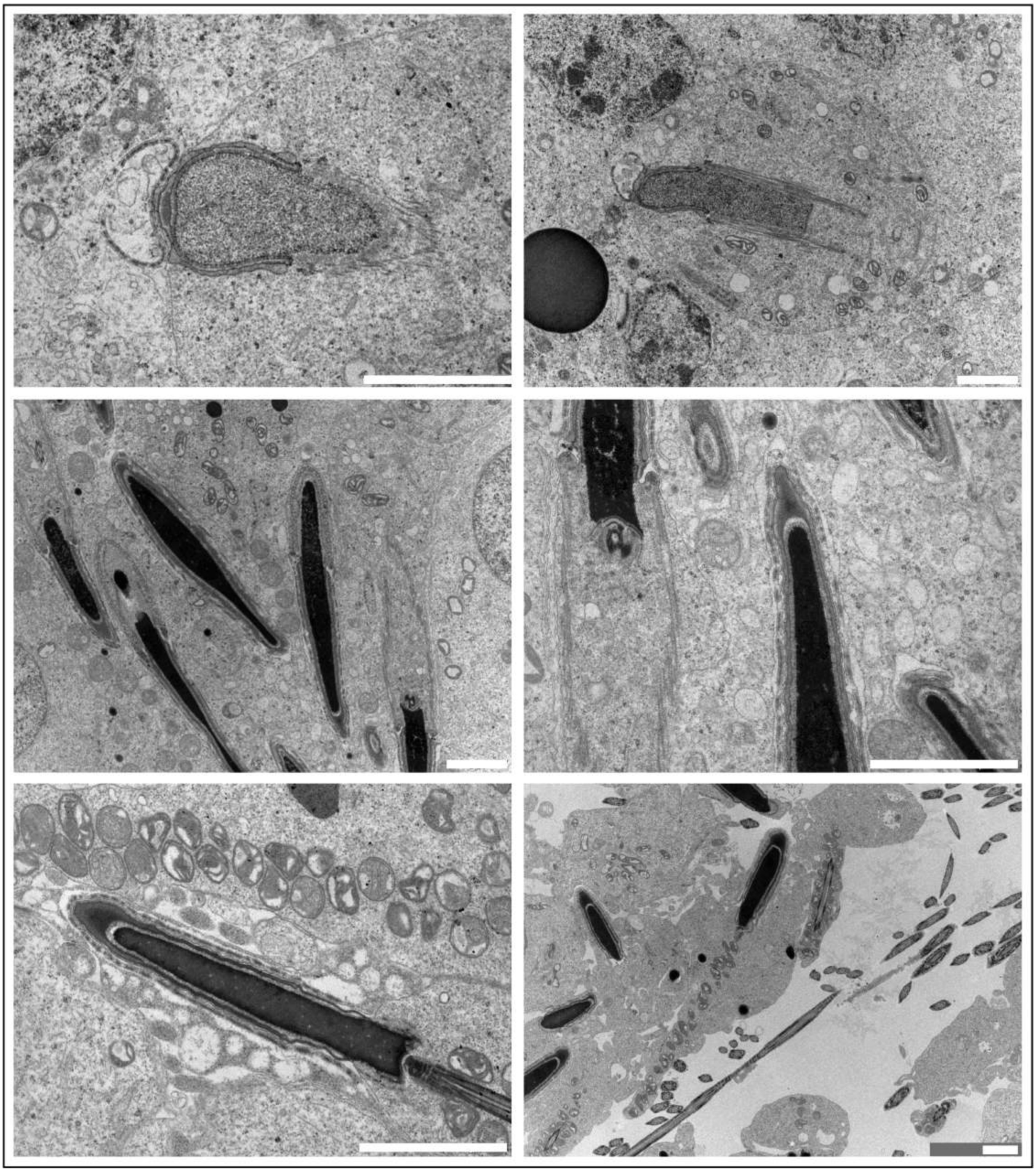

Fig. S8. Transmission electron micrographs of *Prm1-/-* testis. Representative micrographs of *Prm1-/-* seminiferous tubule tissue. Scale: 2 µm.

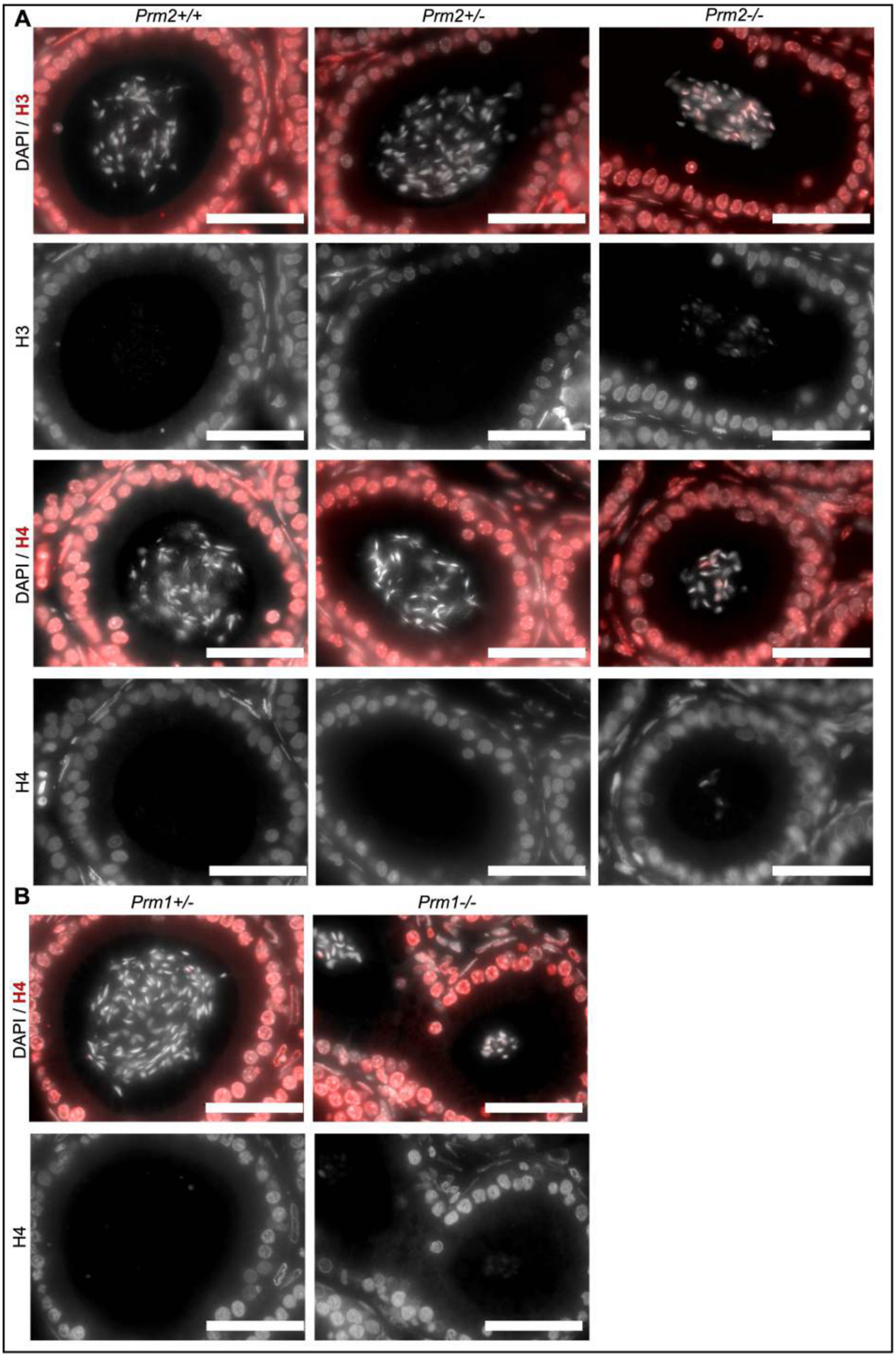

Fig. S9. Immunohistochemical staings against Histones H3 and H4 on caput epididymal sections. (a) *Prm2 +/+*, *Prm2+/-*, *Prm2-l-* sections stained against H3 and H4. **(b)** *Prm1+/-* and *Prm1-l-* sections stained against H4. Antibody staings (red) were counterstained with DAPI (grey) and are shown additionally as grey single channel pictures. Scale: 50 µm.

Development • Supplementary information

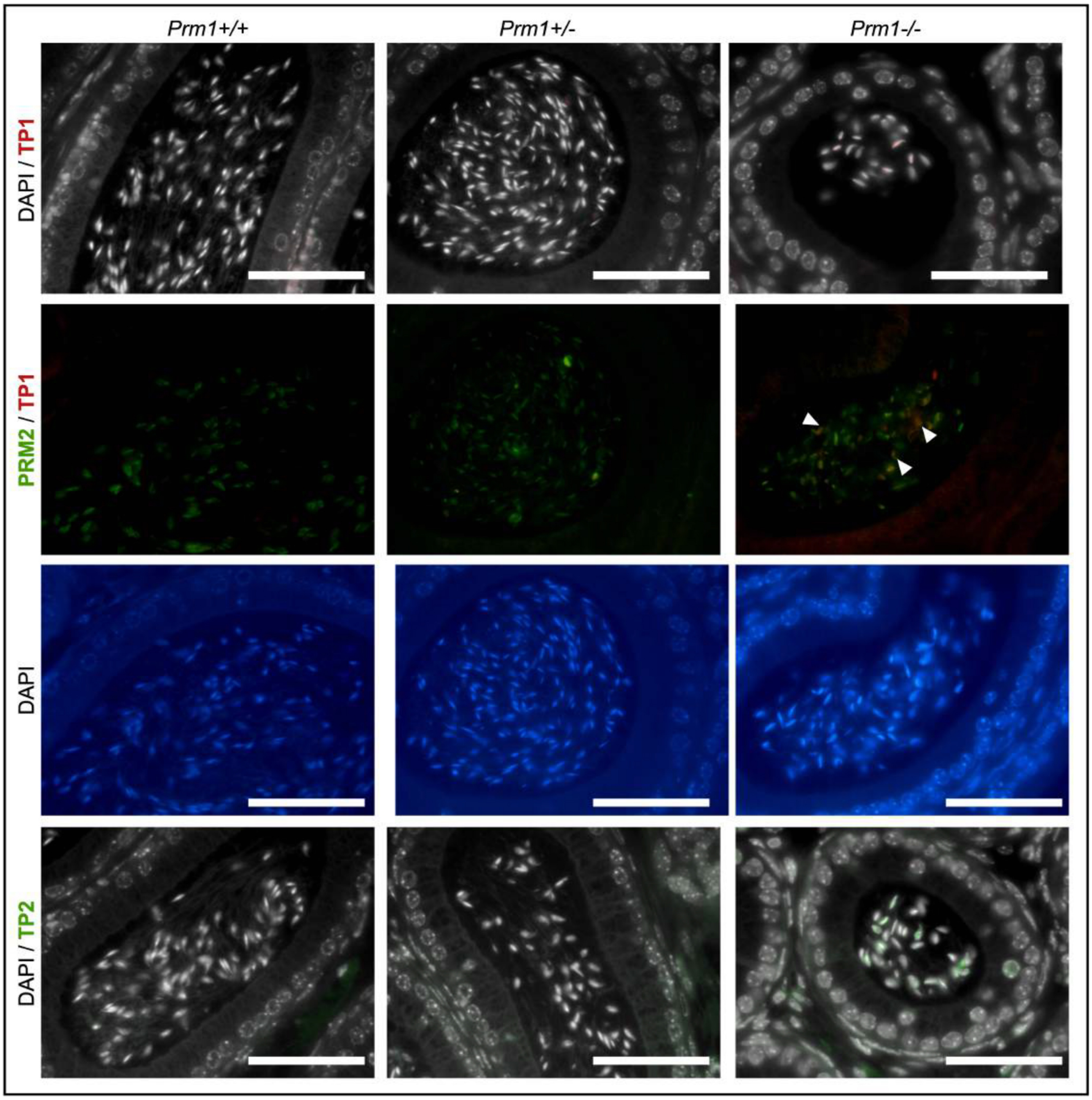

Fig. S10. Representative immunofluorescent staining against TNP1, TNP2 and PRM2. Caput epididymis tissue sections from *Prm1+/+*, *Prm1+/-* and *Prm1-/-* males are shown. Examples of PRM2-TP1 double positive sperm are indicatede with white arrow heads. Scale: 50 µm

Development • Supplementary information

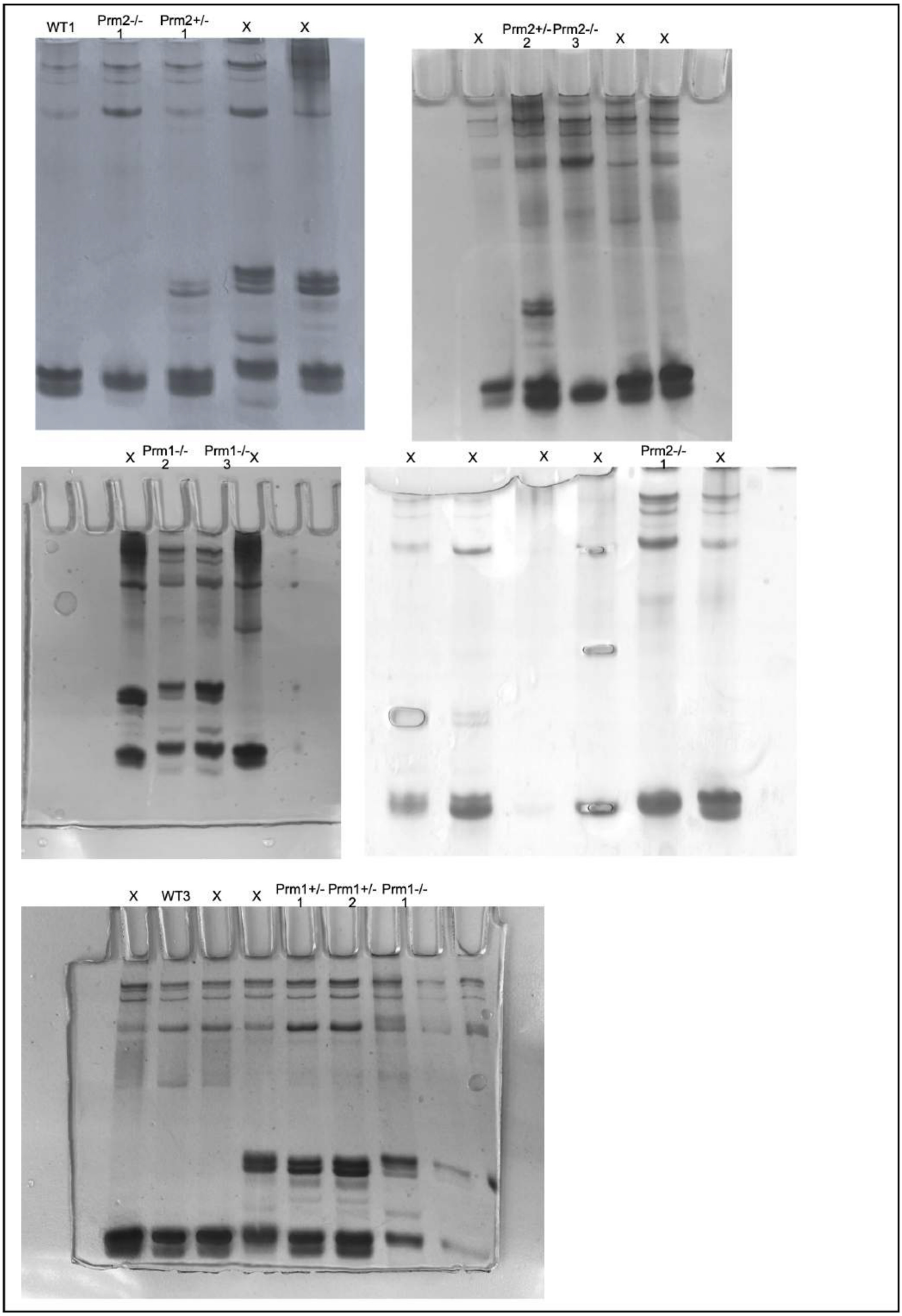

Fig. S11. Acid-urea gels used for band area quantification. Lanes marked with X where not used or part of other studies.

Development • Supplementary information

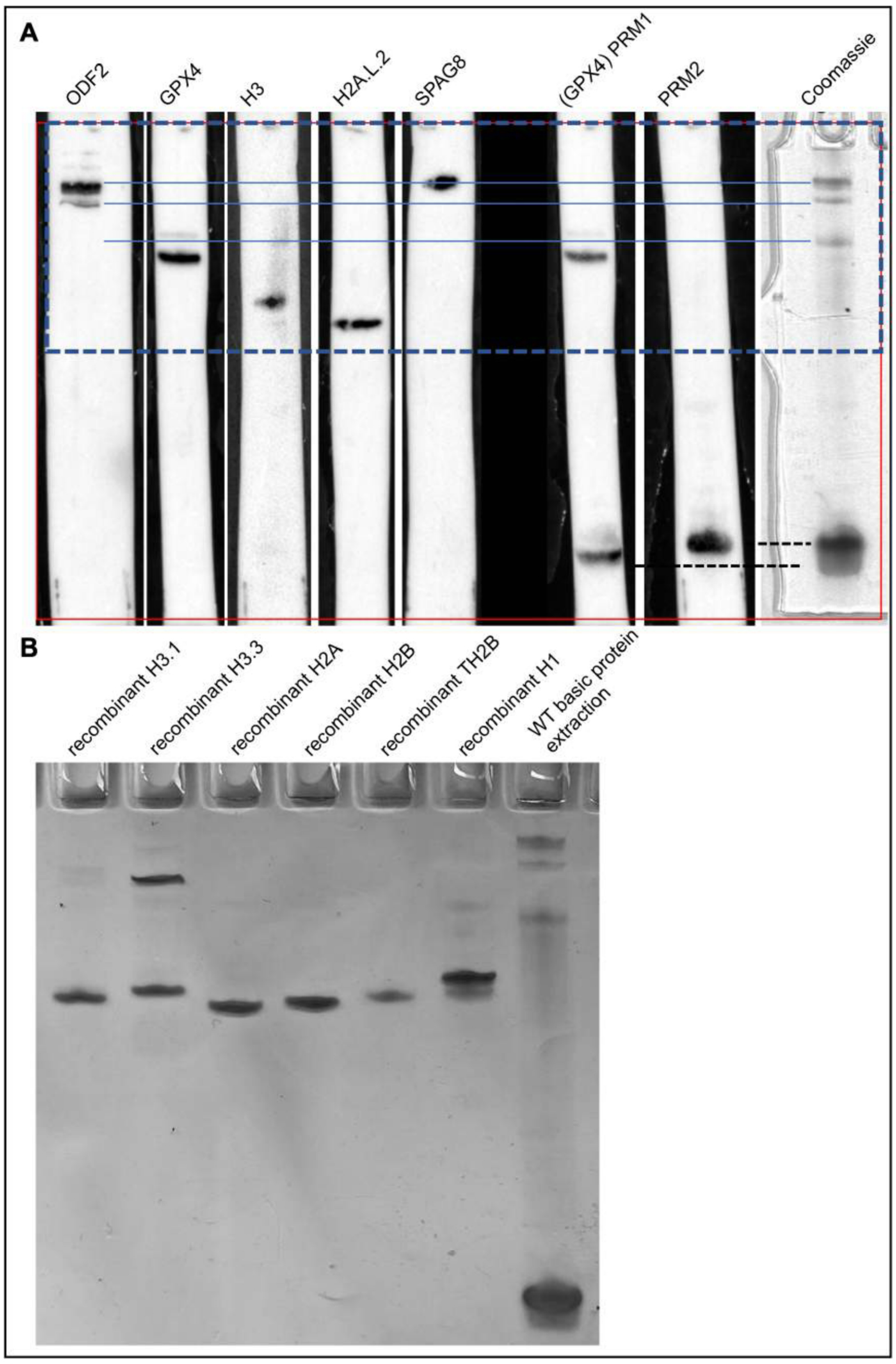

Fig. S12. Identification of proteins isolated from WT sperm for AU-PAGE. (A) Lanes of AU-PAGE gel were loaded with equal amounts (1 million sperm) of nuclear-enriched proteins from pooled WT samples. One lane was fixed and stained with Coomassie, the others were blotted and used for Western Blots. ODF2, GPX4, H3, H2AL2, SPAG8 and both protamines were identified. **(B)** Fixed and Coomassie-stained AU-PAGE gel loaded with equal amounts of recombinant human histone variants. Nuclear-enriched proteins from sperm of pooled murine WT samples were loaded for orientation.

Development • Supplementary information

Table S1. Fertility data Prm1-deficient male mice

genotype	male #	plugs collected	litters born	litter size 1	litter size 2	litter size 3	litter size 4	litter size 5	litter size 6
+/+	1	7	6	9	5	9	7	8	5
	2	5	5	9	9	3	9	8	
	3	5	5	8	3	8	8	8	
	4	5	5	8	4	8	6	8	
	5	5	5	13	8	5	6	9	
	6	5	5	9	1	8	2	5	
	7	5	5	7	8	8	7	4	
	8	5	4	5	8	6	11		
+/-	1	5	4	8	5	5	5		
	2	6	4	6	10	1	6		
	3	5	2	1	2				
	4	5	2	1	2				
	5	5	2	2	8				
	6	5	2	2	1				
	7	5	1	1					
	8	5	1	1					
-/-	1	5	0						
	2	5	0						
	3	5	0						
	4	5	0						
	5	5	0						
	6	5	0						
	7	5	0						
	8	5	0						
	9	5	0						

Table S2. RNA-sequencing data of testis from Prm1-deficient mice

Click here to download Table S2

Table S3. MassSpec data of sperm from Prm1-deficient mice

Click here to download Table S3
