# Supplementary material for: Loss of Prm1 leads to defective chromatin protamination, impaired PRM2 processing, reduced sperm motility and subfertility in male mice
Source: Development. 2022 Jun 17;149(12):dev200330. doi: 10.1242/dev.200330 (PMC9270976; doi:10.1242/dev.200330)
Supplement: Reviewer comments [file develop-149-200330_review_history.pdf]

Loss of *Prm1* leads to defective chromatin protamination, impaired PRM2 processing, reduced sperm motility and subfertility in male mice

Gina Esther Merges, Julia Meier, Simon Schneider, Alexander Kruse, Alexander Christian Fröebius, Gregor Kirfel, Klaus Steger, Lena Arévalo and Hubert B. Schorle
DOI: 10.1242/dev.200330

Editor: Haruhiko Koseki

Review timeline
Original submission: 3 November 2021
Editorial decision: 10 December 2021
First revision received: 20 April 2022
Accepted: 12 May 2022

Original submission

First decision letter

MS ID#: DEVELOP/2021/200330

MS TITLE: Loss of Prm1 leads to defective chromatin protamination, impaired PRM2 processing, reduced sperm motility and subfertility in male mice

AUTHORS: Gina Esther Merges, Julia Meier, Simon Schneider, Alexander Kruse, Alexander Christian Froebius, Klaus Steger, Lena Arevalo, and Hubert B Schorle

I have now received all the referees' reports on the above manuscript, and have reached a decision. The referees' comments are appended below, or you can access them online: please go to BenchPressand click on the 'Manuscripts with Decisions' queue in the Author Area.

As you will see, the referees express considerable interest in your work, but have some significant criticisms and recommend a substantial revision of your manuscript before we can consider publication. If you are able to revise the manuscript along the lines suggested, which may involve further experiments, I will be happy receive a revised version of the manuscript. Your revised paper will be re-reviewed by one or more of the original referees, and acceptance of your manuscript will depend on your addressing satisfactorily the reviewers' major concerns. Please also note that Development will normally permit only one round of major revision.

We are aware that you may be experiencing disruption to the normal running of your lab that make experimental revisions challenging. If it would be helpful, we encourage you to contact us to discuss your revision in greater detail. Please send us a point-by-point response indicating where you are able to address concerns raised (either experimentally or by changes to the text) and where you will not be able to do so within the normal timeframe of a revision. We will then provide further guidance. Please also note that we are happy to extend revision timeframes as necessary.

Please attend to all of the reviewers' comments and ensure that you clearly highlight all changes made in the revised manuscript. Please avoid using 'Tracked changes' in Word files as these are lost in PDF conversion. I should be grateful if you would also provide a point-by-point response detailing how you have dealt with the points raised by the reviewers in the 'Response to Reviewers' box. If you do not agree with any of their criticisms or suggestions please explain clearly why this is so.

© 2022. Published by The Company of Biologists under the terms of the Creative Commons Attribution License (https://creativecommons.org/licenses/by/4.0/).

Reviewer 1

Advance summary and potential significance to field

Here the authors report the generation of mice deficient for Prm1 and proceeded with detailed phenotypical cellular and molecular characterizations of these mice compared to other genotypes (wild-type and Prm1+/-), as well as to Prm2 KO mice.
All the characterizations were performed at the best of professional standards and all the authors' conclusions are supported by the experimental data of high quality.
In conclusion, this manuscript reports important information that should be of interest to a large audience.

Comments for the author

Before publication the authors should pay attention the following points.
1 – Transcriptomic analysis of whole testis from wild-type and Prm1 and Prm2 deficient mice does not sound as a very reasonable approach. Indeed, there is little reason that the impaired protamine incorporation significantly affects gene expression, because it normally happens after a general shut-down of transcriptional activity. This criticism is supported by the fact the authors only identified a very small number of genes that show differential expression.
There is a strong possibility that these genes come from a variable number of contaminating cells and / or from non-germ cells present in the testes studied.
More specifically, Il1b, Ccl5, Saa3, Atp6ap1, Rsad2, Cxcl10, Ifit1, Mmp13, Clec4e, Zghhc could be genes that are expressed in other cell types and hence their enrichment in Prm1 deficient testes, would simply reflects the presence of the corresponding cell types in the analyzed samples. An analysis of the cell type specificity of these genes (in silico) could assess whether they are coming from contaminating cells, and if so, help identifying which type of non-germ cells would be contributing to the studied transcriptome.
2 - The analysis of basic nuclear protein extracts of Prm1+/-, Prm1-/-, Prm2-/- and WT sperm makes more sense. Indeed, a failure in histone-to-Prm replacement could lead to aberrant protein retention or, in contrast, to protein degradation.
Among the enriched proteins, the authors report the presence of HSPA2, which they attribute to stress response. However, they seem to ignore that there is an old study identifying this protein associated with transition proteins (TPs). In this study it was proposed that HSPA2 could act as a chaperone for TPs
(PMID: 17035236).
The accumulation of HSPA2 in Prm1 deficient mature sperms may also suggest an impaired TP unloading from their chaperone, which would remain bound to HSPA2 and stabilize this chaperone. This point could at least be discussed.
Minor points 3 - The abstract is too long and very detailed. The authors should give their general conclusions and avoid to detail all their observations.

Reviewer 2

Advance summary and potential significance to field

Within this study Merges et al have characterised the role of protamine 1 in male fertility using the first homozygous Prm1 knockout mouse. It should be noted that a previous study have characterised the role of PRM1 using a heterozygous Prm1+/- model that was male infertile. Herein the authors have produced a Prm1+/- model which is male subfertile and a Prm1+/+ model which is infertile. As one would expect from a protamine KO mouse, they have shown that the Prm1+/- and Prm1+/+ mice have defects in histone to protamine transition during spermatogenesis resulting in the failure sperm DNA condensation, and in turn increased in DNA damage and compromised integrity of the sperm head. They have also shown that Prm1 deficient mice have sperm motility defects and have reported that PRM1 is required for the processing of PRM2 into its mature form.

Overall, the study is nicely conducted, logically presented and provides a nice characterisation of the role PRM1 in spermatogenesis. It should be noted however most of the functions and mechanisms seen herein recapitulate those seen previously in the Prm1+/- model published by Takeda et al in Scientific Reports and this was not made entirely evident in the current manuscript.

© 2022. Published by The Company of Biologists under the terms of the Creative Commons Attribution License (https://creativecommons.org/licenses/by/4.0/).

For example similar protamine status and DNA damage phenotypes, and similar mechanistic insights that PRM1 is required for PRM2 processing from the precursor to the mature form has been shown in the previous Takeda et al Prm1+/- model. The authors need to more clearly show and explain how their results add onto this previous knowledge, and likewise need to be careful not to overstate the novelty of their findings.

Comments for the author

1. Include ages of male and female mice used for fertility assessments
2. Line 364 – word 'mice' is incorrectly italicised
3. For sperm counting for both OHdG and for counting the number of sperm was a specific seminiferous tubule stage counted? The same stage should be used in case there is any stage specific loss of spermatids.
4. For sperm motility assessments it should be noted that the swim out method will bias towards motile sperm as immotile sperm will be less likely to vacate the epididymis. Back flushing is the gold standard.
5. Histology images in Fig 2d and e need to be white balanced.
6. For the data graphed in Figure 1E and F, N should represent biological replicates (e.g. for each male the average number of pregnancies, or average litter size should be plotted). Statistical analysis should be repeated/corrected for this.
7. Testis weight should always be graphed independent of body size.
8. Line 347 and 335 – your later results show spermatogenesis is affected as histone to protamine transition is a key aspect of spermatogenesis. So your current wording contradicts your later data. Might be good to tweak these lines –
to something like Spermatogenesis is overtly normal in Prm1-deficient mice.
9. In Fig 3F it may help to show the single green channel and the merged channel. The cauda section presented for the cauda for Prm1-/- also looks to be incorrect – the epithelium should be squamous more similar to the cauda image presented for Prm1+/-
10. For figures 4a,b,d and 5b include more detail for the labels on the Y-
axis, and for all figures include definition of p-values and error bars etc in the figure legend.
11. The authors attribute the observed motility defects to oxidative damage does this manifest as structural defects seen in the tails themselves (e.g. via EM)? I note in the Takeda Prm1+/- mice structural sperm tail defects were seen.
More thorough analysis of sperm morphology would be beneficial.
12. Th authors show that the Prm-/- epididymal sperm do not have the apical hook characteristic of mouse sperm. Analysis of sperm head shaping (e.g. on PAS stained sections) on testis sections would be useful to show if this originates due to a failure to form the hook from Stages 10 onwards, or if it is due to degeneration of the nucleus once it reaches the epididymis.
Potential extensions to the study 1. Characterisation of how the head shaping machinery (manchette, acroplaxome etc) is affected by the lack of nuclear condensation would be interesting – e.g via electron micrographs of the seminiferous tubules

Reviewer 3

Advance summary and potential significance to field

This work and manuscript has been performed by very competent authors in the field and represents a very substantial contribution to the understanding of the role of protamine 1.

Using CRISPR-Cas9-mediated generation of Prm1-deficient mice they demonstrate that Prm1+/- mice are subfertile while Prm1-/- are infertile, and correlate with higher levels of 8-OHdG. CMA3 staining is also performed with results that the authors interpret as an indication of the presence of protamine-free DNA in sperm, and claim that is not due to increased histone retention as demonstrated by mass spectrometry of nuclear proteins in Prm1+/- sperm. Using PAGE they also demonstrate that sperm from Prm1+/- and Prm1-/- mice contain a high level of unprocessed, full-length PRM2, suggesting that Prm1 is required for proper processing of PRM2 to produce the mature PRM2.

© 2022. Published by The Company of Biologists under the terms of the Creative Commons Attribution License (https://creativecommons.org/licenses/by/4.0/).

The manuscript reads well and is well focused and referenced. The methods used are also well referenced and described. The work also contributes to the better understanding of the function of the protamine 1 and its relationship to the processing of protamine 2.

I believe that this manuscript will represent an important contribution to the field, opening up new avenues for further investigation.

Comments for the author

I have only some minor comments:
How can the authors completely rule out from the mass spectrometry results in +/- mice are not due to increased histone retention (abstract, lines 33-34). Mass spectrometry results as they have been obtained in the current work (based on PSMs) are not necessarily fully quantitative. Either the appropriate method would be a quantitative MS approach (such as differential labelling and quantification) or a gel based approach. In figure 6C they show a basic protein gel but since no histone standard is included in one of the lanes it is not possible to conclude which of the protein bands may correspond to histones. In fact some of the bands labelled as “non-protamine nuclear proteins” are more abundant or of different mobility in the +/- and -/- lanes as compared to the +/+ lane.
I also wonder whether the measured effects could be due to other concomitant changes that may have been introduced by the CRISPR/Cas9 editing, or the associated methodology. It would be useful if the authors could further comment on this point to completely rule this aspect out.

First revision

<u>Author response to reviewers' comments</u>

Reviewer 1 Advance Summary and Potential Significance to Field:
Here the authors report the generation of mice deficient for Prm1 and proceeded with detailed phenotypical, cellular and molecular characterizations of these mice compared to other genotypes (wild-type and Prm1+/-), as well as to Prm2 KO mice.
All the characterizations were performed at the best of professional standards and all the authors’ conclusions are supported by the experimental data of high quality.
In conclusion, this manuscript reports important information that should be of interest to a large audience.

Reviewer 1 Comments for the Author:
Before publication the authors should pay attention the following points.

1 – Transcriptomic analysis of whole testis from wild-type and Prm1 and Prm2 deficient mice does not sound as a very reasonable approach. Indeed, there is little reason that the impaired protamine incorporation significantly affects gene expression, because it normally happens after a general shut- down of transcriptional activity. This criticism is supported by the fact the authors only identified a very small number of genes that show differential expression.
There is a strong possibility that these genes come from a variable number of contaminating cells and / or from non-germ cells present in the testes studied.
More specifically, Il1b, Ccl5, Saa3, Atp6ap1, Rsad2, Cxcl10, Ifit1, Mmp13, Clec4e, Zghhc could be genes that are expressed in other cell types and hence their enrichment in Prm1 deficient testes, would simply reflects the presence of the corresponding cell types in the analyzed samples. An analysis of the cell type specificity of these genes (in silico) could assess whether they are coming from contaminating cells, and if so, help identifying which type of non-germ cells would be contributing to the studied transcriptome.

Authors response:
Thank you very much for this valuable comment. Indeed, the small number of differentially expressed genes might not represent a solid argument for impaired transcriptional silencing. We have therefore decided to exclude this claim from our manuscript.

© 2022. Published by The Company of Biologists under the terms of the Creative Commons Attribution License (https://creativecommons.org/licenses/by/4.0/).

Regarding the mentioned transcripts (Il1b, Ccl5, Saa3, Atp6ap1, Rsad2, Cxcl10, Ifit1, Mmp13, Clec4e, Zghhc), we made an attempt to validate these via IHC. In IHC stainings no signal for IL1B (and CD45) was detected within the seminiferous tubules on *Prm1-/-* testis sections. Only blood vessels outside the seminiferous tubules stained positive in all samples (*Prm1-/-*, *Prm1+/-* and *Prm1+/+*). Hence, these transcripts might indeed represent contaminating blood cells. These transcripts were slightly higher abundant in *Prm1-/-* testis compared to WT testis, but showed overall low levels of expression.

We have made changes to the results and discussion parts:

<u>Result reads now (lines 235-243):</u>

To address the question, whether transcription is affected upon loss of protamines, we performed transcriptomic and proteomic analyses. 3´-mRNA sequencing of the whole testis revealed that in *Prm1-/-* testis 99 genes are higher and 11 lower expressed, while in *Prm1+/-* testis 28 genes were higher and 39 were lower expressed, both compared to WT testis (**Fig. 5A, Supplementary Material 1**). In *Prm1-/-* testis pathway enrichment for immune related genes (*Il1b, Ccl5, Saa3, Atp6ap1, Rsad2, Cxcl10, Ifit1, Mmp13, Clec4e, Zghhc*) was identified. These transcripts were slightly higher abundant in *Prm1-/-* testis compared to WT testis, but showed overall low levels of expression (**Supplementary Material 1**). These might indicate a reaction to ROS-induced damage or a slight contamination with blood cells.

<u>Discussion reads now (lines 344-345):</u>

Transcriptional profiling of the whole testis revealed only small differences in gene expression in *Prm1-/-* males compared to *Prm1+/-* and *Prm1+/+* males.

<u>(lines. 443-446):</u>

While the crucial species-specific protamine ratio is maintained in *Prm2+/-* sperm, *Prm1+/-* sperm exhibit an aberrant protamine ratio. We demonstrate that *Prm1-/-* and *Prm2-/-* mice display increased histone retention and redox imbalance leading to severe sperm damage, which render males infertile.

Reviewer's comment:
2 - The analysis of basic nuclear protein extracts of Prm1+/-, Prm1-/-, Prm2-/- and WT sperm makes more sense. Indeed, a failure in histone-to-Prm replacement could lead to aberrant protein retention or, in contrast, to protein degradation.
Among the enriched proteins, the authors report the presence of HSPA2, which they attribute to stress response. However, they seem to ignore that there is an old study identifying this protein associated with transition proteins (TPs). In this study it was proposed that HSPA2 could act as a chaperone for TPs (PMID: 17035236).
The accumulation of HSPA2 in Prm1 deficient mature sperms may also suggest an impaired TP unloading from their chaperone, which would remain bound to HSPA2 and stabilize this chaperone. This point could at least be discussed.

Authors response:
This was a valuable comment, we performed IHC using TP and included the results. Further, we discuss the elevation of HSPA2 in this context. In detail we added:

<u>Results section (lines 263-272):</u>

Interestingly, the heat shock-related 70 kDa protein 2 (HSPA2), which is higher abundant in both *Prm1-/-* and *Prm2-/-* samples, has been proposed to function as a transition protein (TP) chaperone in condensing spermatids (Govin et al., 2006). In order to test whether elevated level of HSPA2 affects presence of TPs, we used IF staining against TP1 and TP2 on *Prm1-/-*, *Prm1+/-* and *Prm1+/+* caput epididymis tissue sections (**Fig. S10**). TP1 and TP2 were detected in *Prm1-/-* sperm in the caput, but not in *Prm1+/-* or *Prm1+/+* samples. Higher abundances of HSPA2 in *Prm1-/-* and *Prm2-/-* epidydimal sperm could be indicative for TP-HSPA2 complexes and in turn impaired or incomplete TP unloading. As shown by co-immunofluorescent staining against TP1 and PRM2, the

© 2022. Published by The Company of Biologists under the terms of the Creative Commons Attribution License (https://creativecommons.org/licenses/by/4.0/).

majority TP1-positive *Prm1-/-* caput sperm appeared positive for both proteins.

Material and Methods (lines 516-521):

Slides for IF against TP1 (Abcam Inc., Massachusetts, USA; ab73135; 1:1000), TNP2 (Santa Cruz Biotechnology Inc., Dallas, TX, USA; sc-393843; 1:100), H3 (Abcam Inc., ab1791, 1:1500), H4 (Abcam Inc., ab177840, 1:2000) and PRM2 (1:200) were processed using the VectaFluor^{TM} Duet Double Labeling Kit (Vector Laboratories; DK- 8828) and mounted with ROTI¤Mount FluorCare DAPI (Carl Roth, Karlsruhe, Germany).

Discussion (lines: 351-355):

Interestingly, HSPA2, which was proposed to be a TP chaperone (Govin et al. 2006), was higher abundant in Prm1-/- and *Prm2-/-* sperm. Since we detected slight transition protein retention in *Prm1-/-*, but not *Prm1+/-* caput epidydimal sperm, this might indicate that TP unloading is disturbed in *Prm1-/-* sperm leading to the higher abundant HSPA2 protein level.

Reviewer's comment:
Minor points
3 - The abstract is too long and very detailed. The authors should give their general conclusions and avoid to detail all their observations.

Authors response:
We have completely re-written our abstract which now adheres to the guidelines put forward by "Development".

The abstract now reads 8line 47 -60):

One of the key events during spermiogenesis is the hypercondensation of chromatin by substitution of the majority of histones by protamines. In humans and mice, protamine 1 (PRM1/Prm1) and protamine 2 (PRM2/Prm2), are expressed in a species-specific ratio. Using CRISPR-Cas9-mediated gene editing we generated Prm1-deficient mice and demonstrate, that *Prm1+/-* mice are subfertile while *Prm1-/-* are infertile. *Prm1-/-* and *Prm2-/-* sperm show high levels of reactive oxygen species (ROS)-mediated DNA damages and increased histone retention. In contrast, *Prm1+/-* sperm display only moderate DNA damages. The majority of *Prm1+/-* sperm were CMA3 positive indicating protamine-deficient chromatin. This is not due to increased histone retention in *Prm1+/-* sperm. However, we found that sperm from *Prm1+/-* and *Prm1-/-* mice contain high levels of incompletely processed PRM2. Further, the PRM1:PRM2 ratio is skewed from 1:2 in WT to 1:5 in *Prm1+/-* animals. Our results reveal that PRM1 is required for proper PRM2 processing to produce the mature PRM2 which, together with PRM1 is able to hypercondense DNA. Hence, the species specific PRM1:PRM2 ratio has to be precisely controlled in order to retain full fertility.

Reviewer 2 Advance Summary and Potential Significance to Field:
Within this study Merges et al have characterised the role of protamine 1 in male fertility using the first homozygous Prm1 knockout mouse. It should be noted that a previous study have characterised the role of PRM1 using a heterozygous Prm1+/- model that was male infertile. Herein the authors have produced a Prm1+/- model which is male subfertile and a Prm1+/+ model which is infertile. As one would expect from a protamine KO mouse, they have shown that the Prm1+/- and Prm1+/+ mice have defects in histone to protamine transition during spermatogenesis, resulting in the failure sperm DNA condensation, and in turn increased in DNA damage and compromised integrity of the sperm head. They have also shown that Prm1 deficient mice have sperm motility defects and have reported that PRM1 is required for the processing of PRM2 into its mature form.

Overall, the study is nicely conducted, logically presented and provides a nice characterisation of the role PRM1 in spermatogenesis. It should be noted however most of the functions and mechanisms seen herein recapitulate those seen previously in the Prm1+/- model published by Takeda et al in Scientific Reports, and this was not made entirely evident in the current manuscript. For example, similar protamine status and DNA damage phenotypes, and similar mechanistic insights that PRM1 is required for PRM2 processing from the precursor to the mature form has been shown in the previous Takeda et al Prm1+/- model. The authors need to more

© 2022. Published by The Company of Biologists under the terms of the Creative Commons Attribution License (https://creativecommons.org/licenses/by/4.0/).

clearly show and explain how their results add onto this previous knowledge, and likewise need to be careful not to overstate the novelty of their findings.

Authors response:

As Reviewer 2 correctly stated there has been (as mentioned in the manuscript) previous studies constructing Prm2 deficient mice in order to evaluate the consequences of *Prm1*-deficiency. We emphasize these studies more in our manuscript and put our results into context. We therefore added additional comparative statements as follows:

lines 386-391:

Of note, motility defects have been described in *Prm1+/-* mice generated by Takeda et al (Takeda et al., 2016). They have shown that *Prm1+/-* sperm show a reduction in the mitochondrial membrane potential, which has been associated with reduced sperm motility (Gawlik et al. 2008). Further, scanning electron microscopy revealed various tail deformities in *Prm1+/-* sperm. Here, we saw very similar sperm tail defects both in *Prm1+/-* and *Prm1-/-* sperm transmission electron micrographs.

lines 408-412:

Defects in PRM2 processing were also described for *Prm1+/-* mice published by Takeda et al., histone variant H2A.L.2-KO, transition protein (TPs)-1 and -2 (TP1/TP2)-double KO, TP2-KO and cleaved PRM2 cP2-KO mouse models (Arévalo et al., 2021 preprint, Zhao et al., 2001, Barral et al., 2017, Shirley et al., 2004, Takeda et al., 2016), all of which display fertility problems.

Reviewer 2 Comments for the Author:

Reviewer's comment:
1. Include ages of male and female mice used for fertility assessments

Authors response:
We have added the age of the mice used in fertility assessment in the respective section in Material and Methods

Lines (497-498) now read:

Male mice entered the fertility testing aged between 8 and 13 weeks. Female mice were aged between 10 and 16 weeks.

Reviewer's comment:
2. Line 364 – word 'mice' is incorrectly italicised

Authors response:
We have corrected the format (see line 165).

Reviewer's comment:
3. For sperm counting for both OHdG and for counting the number of sperm was a specific seminiferous tubule stage counted? The same stage should be used in case there is any stage specific loss of spermatids.

Authors response:
8-OHdG positive sperm where not counted on testis sections. Only the sperm in caput and cauda sections were counted. The majority of sperm appeared 8-OHdG positive on *Prm1-/-* testis section staining from spermatid stage 14-15 onwards. This was not the case on *Prm1+/-* or *Prm1+/+* testis sections (see **Fig. 3E**). We did not observe any evident stage specific loss of spermatids.
Since only *Prm1-/-* testicular sperm appear 8-OHdG positive, we do not consider a quantification necessary. In caput and cauda, however, we detected a small fraction of Prm*1+/-* 8-OHdG positive sperm and therefore decided to quantify positive sperm in these tissues (see **Fig. 3F**). Additionally, we think that the number of 8- OHdG positive sperm in the cauda epididymis is most important

© 2022. Published by The Company of Biologists under the terms of the Creative Commons Attribution License (https://creativecommons.org/licenses/by/4.0/).

regarding male mice fertility. We hope that our reasoning as to why we did not count the 8-OHdG positive sperm in the testis makes sense.

Reviewer's comment:
4. For sperm motility assessments it should be noted that the swim out method will bias towards motile sperm as immotile sperm will be less likely to vacate the epididymis. Back flushing is the gold standard.

Authors response:
This is a very good note. However, we would like to point out, that, if there is an experimental bias due to immotile sperm not vacating/being pushed out of the epidydimis during the procedure, in our animals, the percentage of immobile sperm would be even higher. We addressed this potential problem of the assay by adding the following statement

(lines 218-222):

We would like to point out, that sperm from the cauda epididymis was isolated using the swim out assay, which could potentially bias the overall motility assessment towards motile sperm. While the percentage of motile sperm in the *Prm1*+/+ samples is, in the range of published data (Goodson et al., 2011) *Prm1*+/- sperm exhibits significantly lower numbers.

Reviewer's comment:
5. Histology images in Fig 2d and e need to be white balanced.

Authors response:
Thank for pointing this out. The histology images have now been white balanced using the ImageJ macro "White balance correction_1.0" by Vytas Bindokas (Oct 2006, University of Chicago), modified by Patrice Mascalchi (2014, University of Cambridge UK). This was added to the Material and Method section (lines 529-531).

Reviewer's comment:
6. For the data graphed in Figure 1E and F, N should represent biological replicates (e.g. for each male the average number of pregnancies, or average litter size should be plotted). Statistical analysis should be repeated/corrected for this.

Authors response:
We have changed the graph to depict the average litter size and pregnancy frequency per male (see **Fig. 1**). The sample sizes were changed to the number of males used. Additionally, we added the raw data as **Table S1**. The text and figure legends were changed accordingly (lines: 142, 947-951).

Further, we would like to note that during the course of the revision process additional (further backcrossed, N13) males have been used in fertility testing and new data were added. This changes the sample size, but not the significance of the results (see **Fig. 1E-F, Table S1**).

Accordingly, we changed the following statement in the material in methods section:

(line: 475):

Starting from the N4 generation analyses were performed, using male mice aged between 8-13 weeks.

Reviewer's comment:
7. Testis weight should always be graphed independent of body size.

Authors response:
We have changed the graph, legend and text (lines: 151, 953-954) accordingly.

Reviewer's comment:
8. Line 347 and 335 – your later results show spermatogenesis is affected as histone to protamine transition is a key aspect of spermatogenesis. So your current wording contradicts your later data.

© 2022. Published by The Company of Biologists under the terms of the Creative Commons Attribution License (https://creativecommons.org/licenses/by/4.0/).

Might be good to tweak these lines – to something like Spermatogenesis is overtly normal in Prm1-deficient mice.

Authors response:
This is a very good point. We rephrased the respective sections in the manuscript as follows:

Title in the results section now reads (line 149):

Spermatogenesis is overtly normal in *Prm1*-deficient mice

Results (line 156-157):

Apparently, sperm development is overtly normal in *Prm1+/-* and *Prm1-/-* males.

Discussion (lines 330-332):

Spermatogenesis is overtly normal in *Prm1-/-* (and *Prm1+/-*) mice compared to WT mice. Similar results were described for *Prm2-/-* mice, where spermatogenesis appears orderly (Schneider et al. 2016), epididymal sperm however show severe damage.

Reviewer's comment:
9. In Fig 3F it may help to show the single green channel and the merged channel. The cauda section presented for the cauda for Prm1-/- also looks to be incorrect – the epithelium should be squamous more similar to the cauda image presented for Prm1+/-

Authors response:

We changed the picture for the *Prm1-/-* cauda in **Fig. 3F**. Further we included the split channels for a clearer depiction as **Figure S3** (line 184**)**.

Reviewer's comment:
10. For figures 4a,b,d and 5b include more detail for the labels on the Y- axis, and for all figures include definition of p-values and error bars etc in the figure legend.

Authors response:
We have reduced the information contained in **Fig. 4A, B** and **D** to one value and the y-axes were redefined. We have added the definition for the p-values and error bars in the figure legends.

Figure 5B mentioned by the reviewer is the representation of the differentially abundant proteins detected with MassSpec displayed as Venn diagram. This does not contain a y-axis. If **Fig. 6B** was meant (CMA3), we included the same changes.

Reviewer's comment:
11. The authors attribute the observed motility defects to oxidative damage, does this manifest as structural defects seen in the tails themselves (e.g. via EM)? I note in the Takeda Prm1+/- mice structural sperm tail defects were seen. More thorough analysis of sperm morphology would be beneficial.

Authors response:
Thank you for this comment. We looked at TEM of sperm tail cross sections (see **Fig. S7**) and detect structural differences. We added the results

Results (lines 223-228) now reads:

Transmission electron micrographs of flagella cross sections of epididymal sperm showed that a large part of the *Prm1+/-* and *Prm1-/-* sperm do not show the characteristic "9 + 2" microtubule structure (**Fig. S7A-C**). While 98% of *Prm1+/+* sperm flagellar show a "9 + 2" microtubule structure, only 69% of *Prm1+/-* and 54% of *Prm1-/-* sperm flagellar still show a characteristic "9 + 2" microtubule constellation (**Fig. S7D**). Further, severe flagellar membrane damage can be seen in *Prm1-/-* sperm tails.

© 2022. Published by The Company of Biologists under the terms of the Creative Commons Attribution License (https://creativecommons.org/licenses/by/4.0/).

Discussion (lines 386-391) now reads:

Of note, motility defects have been described in *Prm1+/-* mice generated by Takeda et al. (Takeda et al. 2016). They have shown that *Prm1+/-* sperm show a reduction in the mitochondrial membrane potential, which has been associated with reduced sperm motility (Gawlik et al. 2008). Further, scanning electron microscopy revealed various tail deformities in *Prm1+/-* sperm. Here, we saw very similar sperm tail defects both in *Prm1+/-* and *Prm1-/-* sperm micrographs.

Material and Methods (lines 554-555):

Sections used to examine sperm flagella were taken with a scanning electron microscope (Verios 460L, FEI, Eindhoven, Netherlands) equipped with a STEM3 detector.

Reviewer's comment:
12. Th authors show that the Prm-/- epididymal sperm do not have the apical hook characteristic of mouse sperm. Analysis of sperm head shaping (e.g. on PAS stained sections) on testis sections would be useful to show if this originates due to a failure to form the hook from Stages 10 onwards, or if it is due to degeneration of the nucleus once it reaches the epididymis.

Authors response:
To answer this question, we looked at PAS stained testis sections and DAPI stained sperm isolated from testis and included the results (see **Fig. S6**).

Results (lines 210-214) now reads:

Periodic acid-Schiff stainings of seminiferous tubules show that *Prm1-/-* sperm form a hook during spermiogenesis (**Fig. S6A**). The consensus sperm head shapes generated using step 14-16 spermatozoa isolated from testis do not overtly differ between the different genotypes (**Fig. S6B**). This indicates that *Prm1-/-* sperm lose their typical hooked head shape during epididymal transit.

Discussion now reads (lines: 340-343):

Of note, abnormal epididymal sperm head shapes were detected for *Prm1-/-* and *Prm2-/-* sperm (Schneider et al. 2020). Sperm isolated from the testis, however, show normal head shapes, suggesting the altered head shapes are caused by increasing levels of ROS.

Additions to the Material and Methods section have been made (lines: 647-649, 651-658)

Reviewer's comment:
Potential extensions to the study
1. Characterisation of how the head shaping machinery (manchette, acroplaxome etc) is affected by the lack of nuclear condensation would be interesting – e.g via electron micrographs of the seminiferous tubules

Authors response:
This is a very valuable suggestion, which we tried to address. However, our transmission electron microscope (CM10) went out of service and thereafter the scanning electron microscope (FEI Verios 460L) from our collaborator was also broken. Nevertheless, we used the remaining transmission electron micrographs of sperm to compare them to the testis TEM samples. The head shaping machinery appears unaffected. Additionally, we see that the DNA still appears condensed in the final stages of spermiogenesis in *Prm1-/-* mice. Also, the tails seem to be built properly. We were however due to the problems with the TEM and SEM unable run a quantitative analysis. We show the results in the new **Fig. S8** and have added the following to the results:

Results (lines: 228-232) now reads:

In transmission electron micrographs of *Prm1-/-* seminiferous tubules flagella formation and sperm head shaping appear normal, suggesting that sperm tail damages accumulate during epididymal transit (**Fig. S8**). Noteworthy, sperm chromatin appears electron dense indicative of condensed

© 2022. Published by The Company of Biologists under the terms of the Creative Commons Attribution License (https://creativecommons.org/licenses/by/4.0/).

DNA, again suggesting that severe DNA damage and fragmentation accumulate after spermiation.

Reviewer 3 Advance Summary and Potential Significance to Field:

This work and manuscript has been performed by very competent authors in the field and represents a very substantial contribution to the understanding of the role of protamine 1.

Using CRISPR-Cas9-mediated generation of Prm1-deficient mice they demonstrate that Prm1+/- mice are subfertile while Prm1-/- are infertile, and correlate with higher levels of 8-OHdG. CMA3 staining is also performed with results that the authors interpret as an indication of the presence of protamine-free DNA in sperm, and claim that is not due to increased histone retention as demonstrated by mass spectrometry of nuclear proteins in Prm1+/- sperm. Using PAGE they also demonstrate that sperm from Prm1+/- and Prm1-/- mice contain a high level of unprocessed, full-length PRM2, suggesting that Prm1 is required for proper processing of PRM2 to produce the mature PRM2.

The manuscript reads well and is well focused and referenced. The methods used are also well referenced and described. The work also contributes to the better understanding of the function of the protamine 1 and its relationship to the processing of protamine 2.

I believe that this manuscript will represent an important contribution to the field, opening up new avenues for further investigation.

Reviewer 3 Comments for the Author:
I have only some minor comments:

How can the authors completely rule out from the mass spectrometry results in +/- mice are not due to increased histone retention (abstract, lines 33-34). Mass spectrometry results as they have been obtained in the current work (based on PSMs) are not necessarily fully quantitative. Either the appropriate method would be a quantitative MS approach (such as differential labelling and quantification) or a gel based approach. In figure 6C they show a basic protein gel but since no histone standard is included in one of the lanes it is not possible to conclude which of the protein bands may correspond to histones. In fact some of the bands labelled as “non- protamine nuclear proteins” are more abundant or of different mobility in the +/- and -/- lanes as compared to the +/+ lane.

Authors response:
We appreciate this comment, we have addressed the concerns and add further experiments to the manuscript.

While we agree that a quantification based on PSMs would not be sufficient, the mass spectrometric quantifications used here are based on label-free yet reliable precursor ion quantifications: protein abundances are based on summed abundances of extracted ion chromatogram peak areas of protein-group specific peptides. As with all mass spectrometric quantification methods, this does not yield absolute amounts but reliable rankings within and between samples if all LC-MS parameters are kept constant.

In order to further strengthen the results, we performed additional IF staining on cauda epididymal sections and detected H3 and H4 signals in sperm in *Prm1-/-* and *Prm2-/-* samples (**Fig. 5C and Fig. S9**).

The following lines were added to the Results section (lines 252-256):

Increased histone retention was validated using IF staining against H3 on caput epididymal tissue sections (**Fig. 5C, Fig. S9**). H3 was detected in sperm on *Prm1-/-* and *Prm2-/-* sections, but not on Prm1+/-, *Prm2+/-* or WT sections. H4 was detected in *Prm2-/-* sperm, but not in *Prm1+/-*, *Prm2+/-* or WT sperm. Additionally, a weak staining for H4 was detected in *Prm1-/-* sperm.

As for the AU-PAGE we decided to remove the comparison of non-protamine proteins vs. protamines to analyze the protamine content (**former Fig. 6D**). The isolation used is enriched for nuclear

© 2022. Published by The Company of Biologists under the terms of the Creative Commons Attribution License (https://creativecommons.org/licenses/by/4.0/).

proteins, which also contains other sperm-specific basic proteins. To indicate that, we changed the wording from “nuclear protein extraction to “basic protein extraction” throughout the manuscript to be more precise.

The Coomassie stained AU-PAGE is now used to calculate the relative protamine ratios and identifying the pre- PRM2 contents. Since we believe that identification of AU-PAGE bands on basic protein extractions is interesting for other researchers as well we included the identification of some proteins and elaborate on the running behavior of histones (**Fig. S12**) which we added as follows.

Results section (lines 292-296) reads now:

Of note, basic protein extractions are enriched for nuclear proteins, but also contain other basic sperm proteins. We have identified ODF2, GPX4 and SPAG8 to contribute to the prominent Coomassie-stained bands in the upper part of the AU-PAGE (**Fig. S12A**). Noteworthy, canonical histones or testis-specific histone variants run mostly in the middle of the gel (**Fig. S12A-B**).

Note: Due to lack of sample and a validated loading control, we were not able to additionally show increased histone retention using Western Blot. Nevertheless, we believe that validation with IF is suitable.

Reviewer’s comment:
I also wonder whether the measured effects could be due to other concomitant changes that may have been introduced by the CRISPR/Cas9 editing, or the associated methodology. It would be useful if the authors could further comment on this point to completely rule this aspect out.

Authors response:
We have several years of experience with gene editing using CRISPR/Cas based techniques. Several CRISPR/Cas-based mouse models have already been published by us (Schneider et al. 2016, Schneider et al. 2020, Umer et al. 2021, Arévalo et al. 2021). The gRNA’s are all designed using an algorithm predicting low to no off target activity (https://zlab.bio/guide-design-resources). In our experiments the sgRNA’s used for gene editing are tested for function using ES cell culture in advance as elaborated in Schneider et al 2016. Any gRNA yielding non-identified bands upon PCR-analysis will be excluded from further experiments. If, by any chance, off-targets arise, chances are that they have modified/edited DNA on other chromosomes. Such off-targets are not selected for and will segregate away upon backcrossing. We see the Prm1-specific consistent phenotype in all generations analyzed (N4 to N13). We have mapped the transcript reads for *Prm2* and *Tp2* using 3'- RNA-seq in order to address check for off-target events which could influence expression of the *Prm1-Prm2-Tp2* locus, but found no difference. We have included these results in (**Fig. S2**) and added to the results.

Results (lines: 125-126) now read:

Additionally, read mapping to the surrounding *Prm2* and *Tp2* loci was visualized, to exclude local off-target effects, which would not be segregated via backcrossing (**Fig. S2**).

Second decision letter

MS ID#: DEVELOP/2021/200330

MS TITLE: Loss of Prm1 leads to defective chromatin protamination, impaired PRM2 processing, reduced sperm motility and subfertility in male mice

AUTHORS: Gina Esther Merges, Julia Meier, Simon Schneider, Alexander Kruse, Alexander Christian Froebius, Gregor Kirfel, Klaus Steger, Lena Arevalo, and Hubert B Schorle
ARTICLE TYPE: Research Article

© 2022. Published by The Company of Biologists under the terms of the Creative Commons Attribution License (https://creativecommons.org/licenses/by/4.0/).

I am happy to tell you that your manuscript has been accepted for publication in Development, pending our standard ethics checks.

Reviewer 1

Advance summary and potential significance to field

Most of the concerns raised previously have been taken into account and the manuscript has now been much improved. I can therefore recommend this manuscript for publication.

Comments for the author

please see above

Reviewer 2

Advance summary and potential significance to field

Within this study Merges et al have characterised the role of protamine 1 in male fertility using the first homozygous Prm1 knockout mouse. Herein the authors have produced a Prm1+/- model which is male subfertile and a Prm1+/+ model which is infertile. As one would expect from a protamine KO mouse, they have shown that the Prm1+/- and Prm1+/+ mice have defects in histone to protamine transition during spermatogenesis, resulting in the failure sperm DNA condensation, and in turn increased in DNA damage and compromised integrity of the sperm head. They have also shown that Prm1 deficient mice have sperm motility defects and have reported that PRM1 is required for the processing of PRM2 into its mature form.

In the previous review there were concerns raised that the authors had not placed the study in the context of previous studies on PRM1 function, the authors have now sufficiently modified the manuscript to meet these concerns.

Comments for the author

The reviewers have done a good job at addressing all the concerns raised in the original review. I do not think additional revisions are required.

Reviewer 3

Advance summary and potential significance to field

The work contributes to a better understanding of the function of the protamine 1 and its relationship to the processing of protamine 2. I believe that this manuscript will represent an important contribution to the field, opening up new avenues for further investigation.

Comments for the author

The authors have appropriately revisised the manuscript and answered my previous queries. I have no additional comments and beleive the manuscript is appropriate for publication.

© 2022. Published by The Company of Biologists under the terms of the Creative Commons Attribution License (https://creativecommons.org/licenses/by/4.0/).
